# Can lateral tenodesis improve the rotational stability of the ACL reconstruction? A finite element analysis

**DOI:** 10.1371/journal.pone.0293161

**Published:** 2024-02-27

**Authors:** Konstantinos Risvas, Dimitar Stanev, Konstantinos Moustakas

**Affiliations:** 1 Department of Electrical and Computer Engineering, University of Patras, Patras, Greece; 2 École Polytechnique Fédérale de Lausanne, Institute of Bioengineering, Lausanne, Switzerland; Thamar University: Dhamar University, YEMEN

## Abstract

One of the most common knee injuries is the Anterior Cruciate Ligament (ACL) rupture with severe implications on knee stability. The usual treatment is the ACL Reconstruction (ACLR) surgery where the surgeon replaces the torn ligament with a graft in an effort to restore knee kinematics. In case of excessive rotatory instability, Lateral Extra—Articular Tenodesis (LET) can be performed in combination with ACLR. Additionally, LET appears to reduce ACLR graft forces minimizing graft failure chances. However, there are concerns about overconstraining physiological rotation. To gain insight in this controversial topic, we developed an automatic, open-source tool to create a series of Finite Element (FE) models attempting to investigate the interactions of ACLR and LET through simulation. We started by creating a validated model of the healthy knee joint that served as reference for subsequent FE simulations. Then, we created FE models of standalone ACLR and combined ACLR—LET. Each model was assessed by applying a loading profile that resembles the reduction phase of the Pivot—Shift clinical exam. We measured the External Tibia Rotation (ETR), the Posterior Tibia Translation (PTT) of the lateral tibial compartment, and the ACLR graft stress developed around the femoral tunnel insertion site. We observed the following: a) LET reduces ETR and PTT compared to isolated ACLR, b) combined ACLR—LET is more sensitive to LET graft pretension with lower values showcasing performance closer to the healthy joint, c) LET reduces ACLR graft forces for the same pretension values, d) LET exhibits significant overconstraint for higher pretension values. In general, these findings are in agreement with relevant clinical studies and accentuate the potential of the developed framework as a tool that can assist orthopaedists during surgery planning. We provide open access for the FE models of this study to enhance research transparency, reproducibility and extensibility.

## Introduction

Knee joint injuries have become a commonplace in demanding physical activities and competitive sports, such as soccer, basketball, volleyball and skiing [1, 2]. One of them is the typical Anterior Cruciate Ligament (ACL) injury where the native ACL is partially or totally ruptured. The ACL’s primary biomechanical role is to restrain Anterior Tibial Translation (ATT). It also acts as a secondary rotational stabilizer, reducing excessive Internal Tibial Rotation (ITR). Consequently, such an injury reduces the physical capability and deteriorates the quality of life of the injured population with further implications on their psychological condition. Moreover, ACL usually demonstrates limited healing capability which is drastically eliminated in total rupture. Therefore, a treatment to restore knee kinematics is of paramount importance, especially in the case of professional athletes.

The type of treatment is usually selected based on the long term objectives and whether the injured person wishes to resume demanding physical activities in the near future. Assuming this requirement, a surgical treatment to the injury is the standard approach, and the Anterior Cruciate Ligament Reconstruction (ACLR) has emerged as the prevalent surgical method. ACLR is a demanding surgical procedure where a plethora of additional parameters needs to be determined. Although ACLR exhibits excellent functional restoration of ATT, the re-establishment of rotational knee stability is still questionable. This type of instability is also related to ACLR graft rupture and early progression of Osteoarthritis (OA) [3]. Towards this direction, additional surgery techniques that are often performed along ACLR have been developed. Lateral Extra-Articular Tenodesis (LET) is a renascent surgical approach that aims to enhance knee rotational stability and is usually performed along ACLR, especially on athletes that perform activities where cutting and pivoting are dominant [3–6]. From a clinical perspective, LET surgery usually involves drilling a tunnel through the femur bone, posterior and superior to the femoral attachment of Lateral Collateral Ligament (LCL). The graft is usually a 10 mm wide strand stripped of the Iliotibial Band (ITB) [3, 7, 8]. The surgeon passes the graft beneath the LCL, applies a pretension load and fixes it at a given knee flexion angle.

Apart from the specific surgical parameters and techniques, the surgeon’s ability and experience are critical factors that affect the outcomes of surgeries, such as ACLR and LET. Also, the optimal combination of the surgery-related criteria for each individual patient is not known in a pre-surgical setting. Rather, the surgical results are evaluated when each subject resumes physical activities which impose high loads to the reconstructed knee joint. Although *in vivo* evaluation of ACLR has been applied to clinical trials [9], clinicians usually resort to clinical exams such as the Lachman and Pivot—Shift (PS) tests for surgery assessment. The former performs outstandingly well when assessing ATT [10]. On the other hand, the latter is better suited for assessing anterolateral knee instability. However, these tests depend on the clinician’s experience and feature high sensitivity and specificity [10–12].

All these pitfalls have led to the emergence of biomechanics simulations as a valuable alternative tool, available to clinicians, engineers and researchers. These simulations are based on computational numerical techniques with the Finite Element (FE) method being one of the most prominent. In the case of ACLR, several FE studies can be found in literature. A common starting point is the creation of a subject-specific FE model [13] based on Magnetic Resonance Imaging (MRI) and Computational Tomography (CT) data. Then, this model is validated in terms of joint kinematics [14–16] and material properties and serves as baseline for subsequent research scenarios [17, 18]. The majority of the FE ACLR studies simulated clinical exams, such as the Lachman Test (or Anterior Drawer test) [14, 19–23] and the PS test [20, 22, 24–26]. Simulations of dynamic physical activities, such as gait, can also be found [16, 23]. The results are usually knee joint kinematics or internal forces (stresses) subject to the adopted material models and compared to clinical data for model validation. Many studies evaluated restoration of knee kinematics and graft loading based on anatomical tunnel placement [16, 17, 21–23, 27]. Other ACLR studies focused on assessing the effect of ACLR parameters, such as graft dimensions, graft pretension and graft fixation angle [18, 19]. On the other hand, and to the best of our knowledge, the literature lacks systematic FE studies that attempt to model and assess the combined ACLR—LET surgery.

As it was mentioned, LET is performed when the ultimate objective is to restore knee rotational stability, albeit caution is required to avoid excessive rotational constraint, a situation that can lead to increased knee stiffness and loading to the lateral knee structures [28]. These concerns along with the absence of relevant FE studies served as motivation for our work. Moreover, most of ACLR FE studies do not provide open access to their models and these are also generated using commercial software tools. In an attempt to confront these challenges, we performed a FE study to investigate the presumed advantages and drawbacks of the combined ACLR and LET surgery in a simulation setting. Our objective was to develop a pipeline that can create multiple FE instances of the knee joint each featuring different ACLR and LET surgical parameters. Then, to use these models in what-if scenarios to confirm or contradict clinical observations or investigate new surgical approaches overcoming the complexity of clinical trials and experiments.

Towards this direction, we expanded our previously published workflow [18] to encompass LET modeling employing open source software tools. The FE model assembly is performed through a custom Python module that we made publicly available. We created a validated FE model that represents the healthy knee joint and serves as the baseline for ensuing ACLR—LET simulations. These include variations of the reconstructed knee in terms of adopted surgery (standalone ACLR or combined ACLR—LET) and graft pretension. We also adopted a loading profile that is capable to produce the PS effects in a simulation setting. We used this profile to assess ITR and ATT restoration and graft stresses after ACLR and LET surgery. We expected to replicate the following clinical observations: 1) LET contributes to restoration of rotational stability when performed along ACLR, 2) LET moderately reduces the ACLR graft stresses, 3) large LET graft pretension can lead to knee over-constraint, and 4) the relationship between ACLR and LET graft pretension has an impact on knee kinematics. We compared our results with clinically relevant studies to evaluate whether our modeling approach can effectively capture the effects of the real-life surgery. Finally, we share all created FE models with the research community aiming towards reproducibility, extensibility and enhanced research translucence.

## Materials and methods

**Fig 1:**
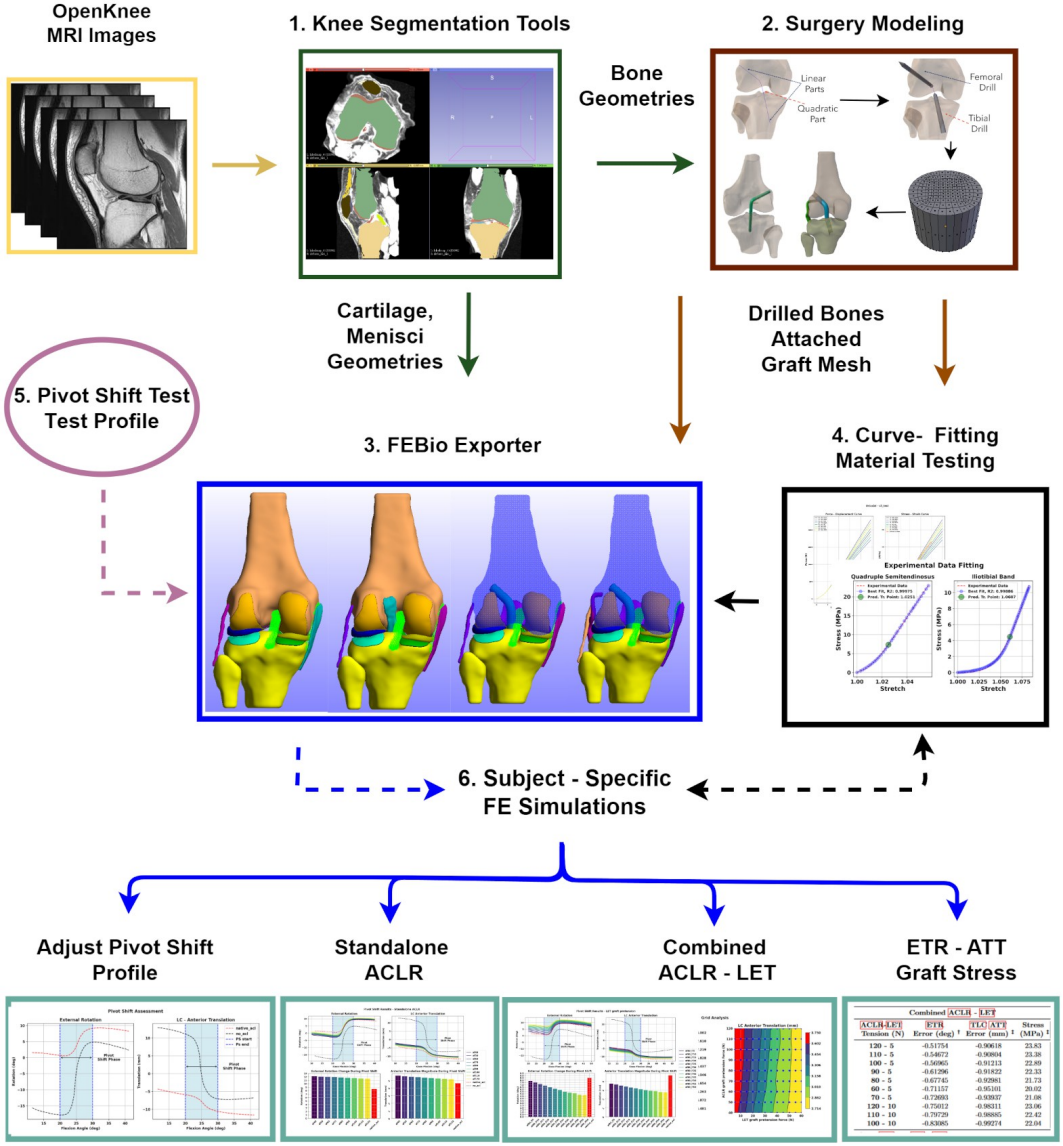
Overview of the proposed workflow. 1. The workflow starts with segmentation of MRI data to obtain subject specific anatomical geometries. 2. We developed the “Surgery Modeling” tool that utilizes Blender scripting to model the ACLR parts (drilled bones and grafts). 3. We created the “FEBio Exporter” tool to assembly subject—specific FE models of the healthy, injured and ACLR knee and to solve them using the FEBio software. 4. We adjusted graft material properties based on experimental data. 5. We developed a suitable loading profile in an effort to simulate the standard physical clinical exam of PS. 6. We performed a series of FE simulations to compare the standalone ACLR versus the combined ACLR—LET surgery techniques in terms of kinematics restoration and ACLR graft stress development.

In Fig 1, we present an overview of the adopted methodology and the distinct building blocks of the proposed workflow. In summary, we utilize MRI data acquired from the OpenKnee (s) project [29] and segment them to acquire surface representations of the anatomical structures. Subsequently, we employ open—source custom tools to create suitable volumetric meshes for the ligaments, cartilages, and menisci [30]. Additionally, we created the “Surgery Modeling” tool to model the ACLR surgery procedure. This tool utilizes Blender scripting, to create the drilled bones and graft geometries. The cornerstone of this work is the “FEBio Exporter” tool, which we utilize as an interface to build all FE models, and execute them with the FEBio solver [31]. Moreover, we developed scripts for experimental curve-fitting to estimate material parameters for the selected grafts. Also, we experimented with boundary conditions representing the reduction phase of the PS clinical exam. We applied those in multiple FE simulations to create a parameter sensitivity study and investigate the impact of LET surgery on knee kinematics and ACLR graft stress.

### Surgery modeling

Initially, we utilized the “Surgery Modeling” tool that was developed and described in detail in our previous work [18]. We modeled different versions of ACLR procedures, namely the standalone Single Bundle (SB) ACLR and the combined ACLR—LET surgeries. The bones were acquired by the OpenKnee (s) database and correspond to the subject “oks003”. The SB ACLR graft features a radius of 4 mm and is passed through the bones close to the attachment sites of the native ligament [32]. The ITB graft is 10 mm wide close to its attachment to the tibia bone to resemble the ITB strip in relevant clinical surgeries [3, 7, 8]. A femoral tunnel with diameter of about 5 mm was created for LET graft fixation. Using the tool’s trajectory planning curve the graft was passed superior to the proximal LCL and through the femoral tunnel. The graft’s distal part was considered fixed with the tibia. We should emphasize that a segmented geometry of the ITB was not available, thus, we decided to place the proximal end of the ITB close to the Gerdy’s tubercle area of the tibia bone [3].

### FE model assembly

In this section, we describe the workflow for creating the FE models. For each simulation scenario we describe the used geometries, adopted materials, enforced contact models and applied boundary conditions. We developed the “FEBio Exporter” tool (Fig 1) for automated creation of FE models suitable for the FEbio solver. This provides us the ability to create multiple instances of models, each featuring different properties. Therefore, these models can be efficiently deployed in sensitivity analyses to investigate what-if scenarios, as in this work.

**Fig 2:**
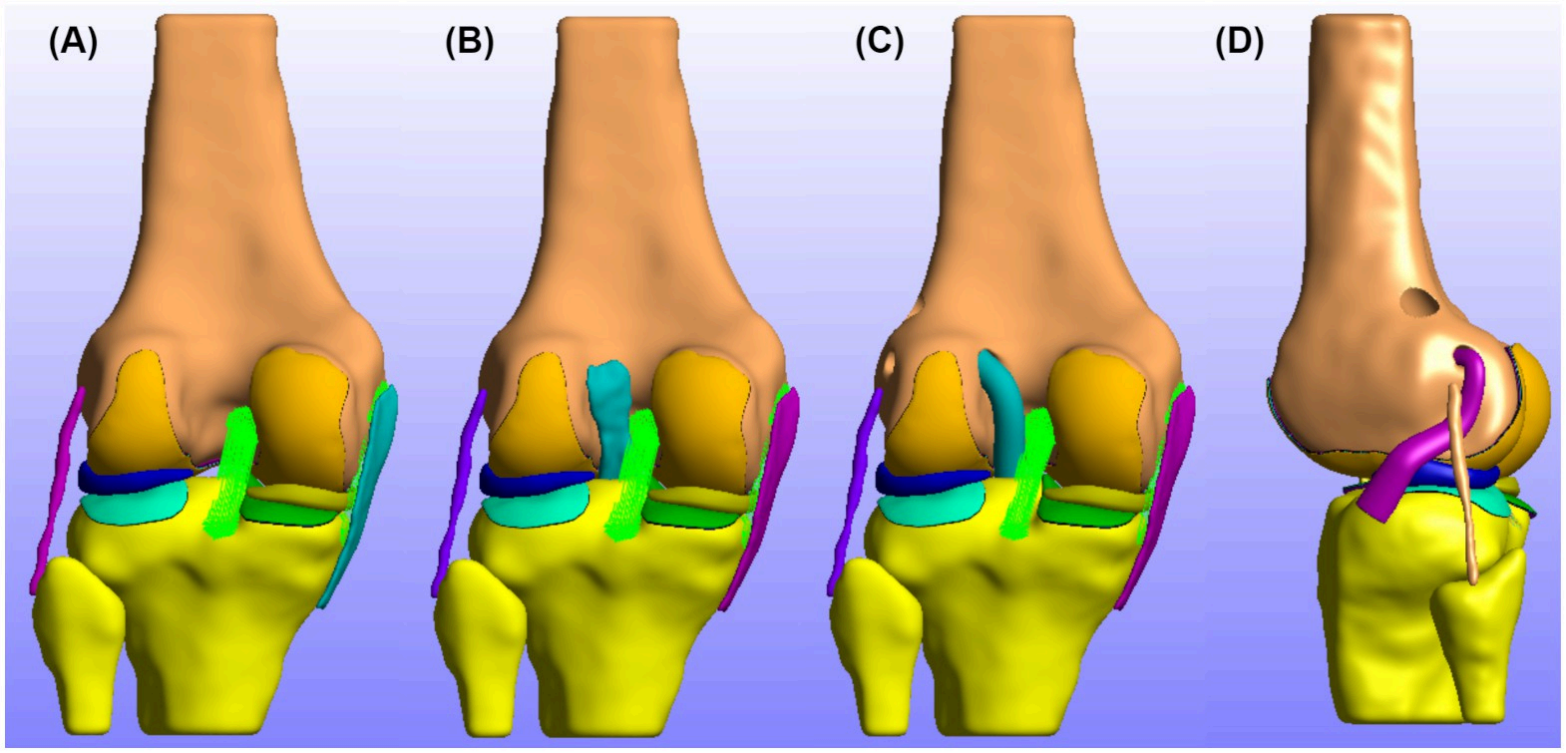
Developed FE models. In this figure we present form left to right the developed FE models used throughout this study. A: No—ACL (injured) knee FE model. B: Native—ACL FE model. C: Standalone SB ACLR FE model. D: Combined ACLR—LET FE model.

During this study, we created the following FE models: a) No-ACL model, that represents the injured joint, b) Native-ACL model, which represents the healthy knee, c) the ACLR—SB model that corresponds to standalone ACLR surgery, and d) the combined ACLR—LET model. The developed models are presented in Fig 2.

#### Geometries and material properties

In the scope of this study, we adjusted a validated FE model used in our previous work [18]. The bone geometries were acquired directly from the Openknee (s) database in the case of the healthy and injured knee joint FE model. In the case of standalone ACLR and combined ACLR—LET the bones were modified by applying the “Surgery Modeling” as described above. The ACL, Medial Collateral Ligament (MCL), and LCL were represented by volumetric meshes, that were generated using the Tetgen software [33]. The material properties were defined based on the corresponding validated OpenKnee FE model. The Posterior Cruciate Ligament (PCL) ligament was modeled as a bundle of nonlinear tension-only springs with a force—displacement curve that was calibrated and validated in our previous study [18]. To enhance the validity of the model, we reproduced the anterior laxity experimental tests of the OpenKnee (s) project in a FE analysis and adjusted accordingly the pre—strain value of the ACL ligament. The meshes and material properties for the femoral and tibial cartilages as well as the menisci were identical as in our previous model [18].

The grafts were modeled with an appropriate hyperelastic transversely isotropic Mooney—Rivlin material featuring an uncoupled deviatoric and volumetric behavior. The numeric values for the model parameters were defined based on curve-fitting on experimental data. We extracted the latter from studies performing uniaxial tests on the quadruple semitendinosus tendon and iliotibial band tissues [34, 35]. These are commonly used as harvesting sites among orthopedic surgeons during ACLR and LET [3, 6, 8]. Furthermore, we performed a series of uniaxial simulations to decide the optimal number of elements (mesh independence test) and proper values for the model parameters, such as the Bulk’s modulus. We provide a detailed description for determining the material properties in the S1 File.

#### Boundary conditions

The majority of the contact modeling parameters between each anatomical structure were adopted from our previous model. We used the same sliding—elastic contact algorithm to model the interaction between the LET graft and the LCL, femur and lateral meniscus. The knee joint was modeled as a combination of three cylindrical joints creating a four—link kinematic chain. The femur and tibia coordinate systems were adopted by the OpenKnee (s) project [15, 29]. The knee flexion was prescribed as a rotation between the rigid bodies of the first cylindrical joint and the loads were applied to the femoral reference frame.

Regarding the PS loading scenarios, in the case of the No-ACL and the Native-ACL models the following simulation steps are defined: 1) Use the Pre—Strain FEBio plugin [36] to apply in-situ strain in the ligament materials, 2) Apply the PS profile, where the tibia rigid body is fixed in all Degrees Of Freedom (DoFs) and the femur is free in all directions.

Subsequently, in the case of ACLR—SB models, the simulation steps are: 1) Pre-Strain plugin application, 2) knee flexion up to the desired graft fixation angle, 3) application of graft pretension force at the free end of the graft and then, graft fixation (fix the free end nodes to the tibia rigid body reference), 4) knee full extension, 5) application of the PS profile.

Finally, for the ACLR—LET simulations, we additionally implemented the following simulation steps after the ACLR graft fixation step: 1) knee flexion up to the LET graft fixation angle and, 2) LET graft pretension and fixation. It is a common practice to perform LET after ACLR [3, 37]. All other steps were the same as above. For all ACLR knee models, the tibia is fixed in all DoFS. The femur is fixed in the anterior—posterior and lateral—medial displacement DoFs and internal—external rotational Degree Of Freedom (DoF) during graft fixation and prior to PS loading, to avoid introduction of displacement or rotational bias to the model. When the PS profile is applied the femur is set free similar to the No-ACL and the Native-ACL simulations.

#### Graft pretension

Regarding graft pretension, we started by defining the orientation of the tunnel where the graft was going to be pulled through. This was the tibial tunnel in the case of SB ACLR surgery and the femoral tunnel for LET, respectively. The orientation was specified using the landmarks that define the surgery planning trajectory inside the respective bone tunnel. Then, a forward simulation was performed where a knee flexion was prescribed using a ramp function in the range of 0°–90° with a step of 10°. The femur was free to move, whereas the tibia was fixed at all DoFs. At each step, we extracted the quaternion that described the femoral rotation. Using Spherical Linear Interpolation (SLERP), we could estimate the quaternion corresponding to any desired fixation angle and the corresponding rotation matrix. This rotation matrix was then multiplied with the initial unit vector (tunnel orientation) to approximate the direction of graft pretension force at the desired fixation angle. Then, the direction vector was scaled by the desired pretension force magnitude. The tension was applied using a ramp function. A rigorous description of the process is provided in S1 File to ease reproducibility.

In our study, the knee flexion angle during graft fixation was set to 25° for ACLR [11] and 30° for LET graft fixation [7, 8, 28]. At the end of the graft pretension step, a specific set of nodes that were inside the tunnel were connected to the corresponding bone mesh, signaling the graft fixation phase. During the subsequent simulation steps, the displacement of these nodes was identical to the displacement of the corresponding rigid bone.

#### Clinical pivot shift

In the clinical setup of the PS exam, the knee is usually flexed at between 10°–20° and a combined load of internal torque, valgus stress and usually anterior force are applied to the tibia. This is the “subluxation phase” of the PS. A test is marked as positive if an anterior subluxation of the lateral tibia plateau is apparent [38]. Subsequently, as the orthopedist flexes the knee a reduction of the loaded tibia occurs approximately between 20°–40° [10, 11, 39]. In general, the knee kinematics exhibit two key features during the “reduction phase” of an effective PS test: a) n abrupt anterior translation of the femur relative to the tibia, and b) a simultaneous excessive internal femoral rotation. These correspond to a posterior tibia translation and external rotation, respectively. There can be multiple PS profiles that may lead to the above described PS kinematics [10].

**Fig 3:**
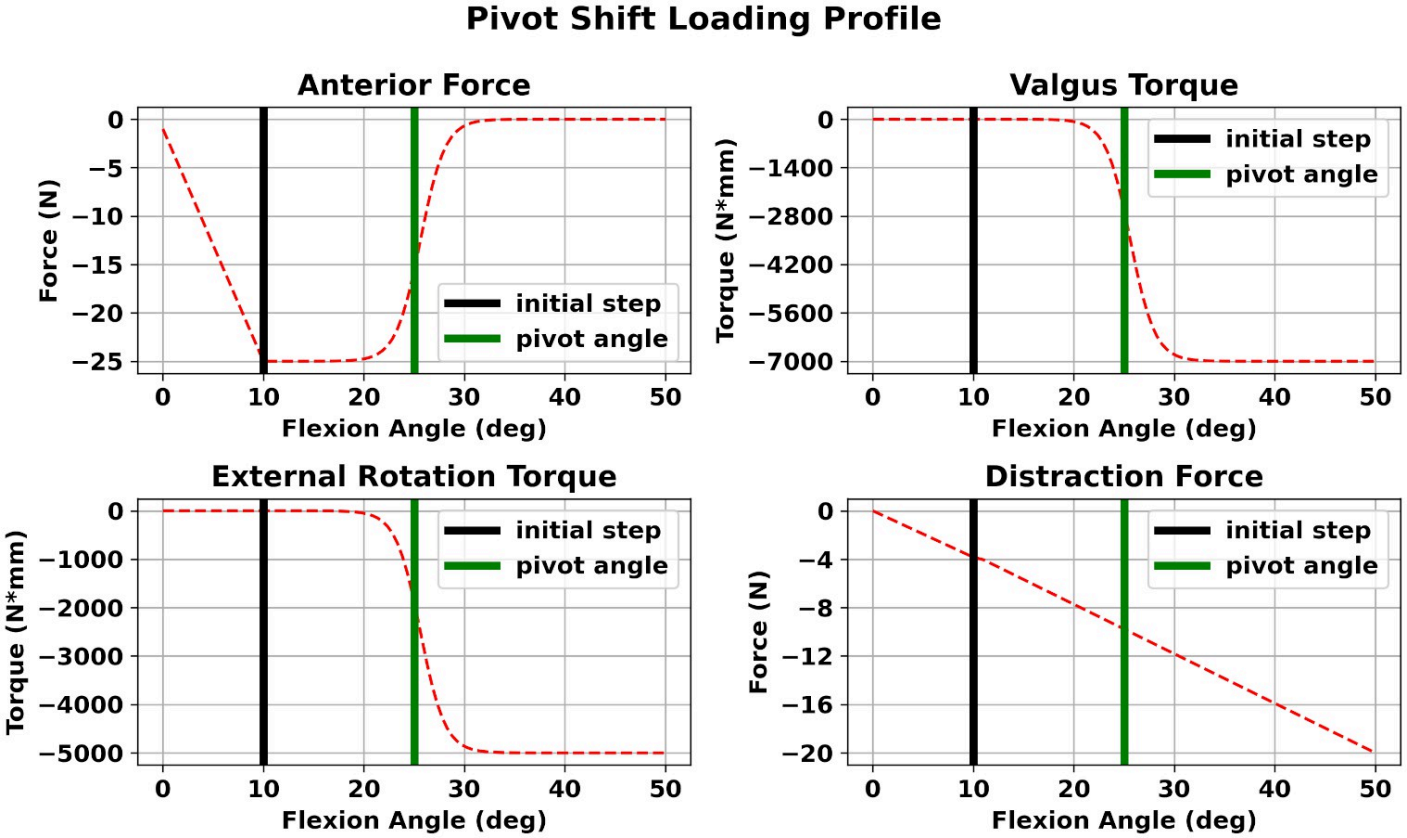
PS loading profile. Starting from left to right and from top to bottom, we observe the applied loads on the femoral reference frame to simulate the PS movement. A posterior force of 25 N is applied up to an initial flexion angle of 10° to induce the subluxation phase. As the knee flexes, an anterior force is applied to model the reduction phase. Additionally, we apply a femur varus torque of 7 Nm and an internal femur moment of 5 Nm. These forces are implemented using sigmoid functions and are applied as the knee flexes from the initial angle of 10°. The transition point of the sigmoids is set to 25° (green vertical line). Finally, we apply a compression force to engage contact between the tibial and femoral cartilages and assist the FEBio solver. The profile is a modified version of the profile in [11].

In this study, we adopted and modified a static loading profile from a robotic clinical study [11]. The applied profile is depicted in Fig 3. Starting from left to right and from top to bottom, we applied a posterior force of 25 N on the femur up to an initial step of 10° of knee flexion. For the rest of the simulation we applied an anterior force of 25 N. Furthermore, we applied a varus torque of 7 Nm and an internal torque of 5 Nm. The anterior—posterior force, and varus and internal rotation torques were implemented using sigmoid functions. The transition area of each sigmoid corresponds to a knee flexion angle in the range of 20°–30°. This is evident by observing that the monotonic abrupt change of each loading curve is specifically defined in the above range. Finally, we applied a compression force of 20N to bring the cartilages into contact and assist the FEBio solver. All forces were applied on the femur reference frame. The adopted PS profile can be considered as a “reduction” PS, since the PS motion occurs during the knee flexion phase.

#### FE case studies

In advance of the results section, we think a comprehensive description of the FE case studies that we conducted in the scope of this work is imperative to assist the reader. Initially, we validated the behavior of the healthy ACL model by reproducing the anterior drawer test form the OpenKnee (s) project and adjusting the ligament’s *in situ* strain. Since this strain affects joint mechanics we envisioned that a proper pre—strain would enhance our model’s validity [36]. The residual strain was applied by defining a proper fiber stretch along the material’s fiber direction. We focused on ATT and used linear regression to fit a line to the experimental values. This line served as the baseline for assessing subsequent FE simulations where we reproduced the same experiment using the Native—ACL FE model that includes the intact ACL. In each simulation we altered the fiber stretch applied to the ACL material model, we applied the same loading profile, and finally we estimated the ATT. We adopted the Mean Squared Error (MSE) as our metric. The optimal fiber stretch corresponded to the model that exhibited the lowest MSE between the simulated ATT and the reference line.

**Fig 4:**
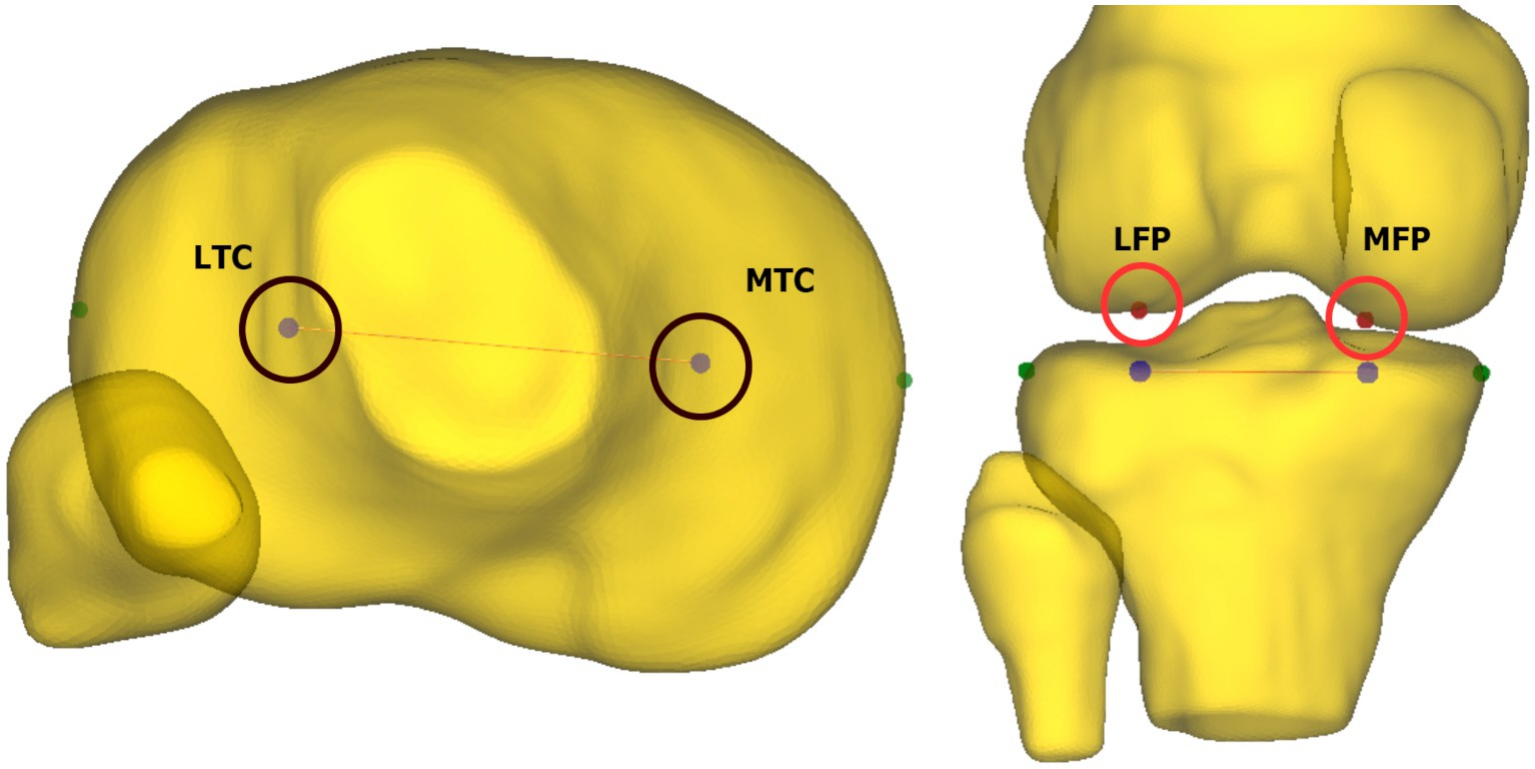
Femur projections of LTC and MTC points. In this figure, we illustrate the estimated LTC and MTC points and their projections on the femur mesh. These points are taken at 25% and 75% of the tibia width [40]. The LTC projection on the femur (LFP) is used to measure the PTT during the PS simulation.

Subsequently, we performed a FE simulation to assess the ability of the adopted PS scenario to produce the characteristic kinematic features of the clinical PS test. Towards this objective, we applied the profile to the No—ACL and the validated Native—ACL models and compared their performance. For this and the ensuing simulation scenarios, the variables of interest were External Tibial Rotation (ETR) and Posterior Tibial Translation (PTT) of the Lateral Tibial Compartment (LTC) during the reduction phase of the adopted PS. Regarding PTT we adopted the following methodology. We identified the most lateral and medial aspects of the tibia plateau points. Their distance is the tibia plateau width. Then, the Medial Tibial Compartment (MTC) and LTC center points are estimated at 25% and 75% of the tibia width and along the line connecting the two aspects [11, 40]. Afterwards, we projected these points on the femur surface by estimating the vertices of the femur mesh that are closest from the line passing through each center point and with a direction along the z-axis. These points are presented in Fig 4.

Moreover, we compared the performance of standalone ACLR surgery and specific cases of ACLR—LET under the same PS profile and by applying different graft pretension values for both cases. We considered that the LET graft pretension is related to the degree of ETR reduction. Also, we wished to investigate whether the combined surgery provides better results for the same ACLR graft pretension as in the isolated surgery. Finally, the ACLR graft pretension is related to the developed graft stresses around the femoral tunnel. In both techniques the adopted tension values were found in literature. These correspond to 80 N for the ACLR graft and 40 N for the LET graft [6, 7, 40, 41]. The ACL graft tension was altered by a factor of ±50%. The LET graft tension values ranged from 5 N (“minimal tension”) up to +50% of the literature value.

Finally, we performed a sensitivity analysis for assessing how different combinations of ACLR and LET graft pretension values affect the model’s performance in restraining the ETR andPTT. We wished to assess the interrelationship between different pretension values of both grafts in reducing kinematic laxity and ACLR graft stress. Therefore, we estimated the developed stress around the femoral tunnel having already gained confidence in our graft mesh resolution.

## Results

In this section, we present the results for each simulation case starting from the standalone ACLR surgery. We provide the graphs illustrating the evolution of ETR and PTT during the PS simulation step for each case. We also present the magnitude of these variables during the PS movement in a bar plot to provide an intuitive comparison between the reconstructed knee models and the healthy knee joint. The validation of the Native—ACL model as well as a detailed assessment of the adopted PS profile and its impact on knee kinematics are provided in the S1 File.

### Standalone ACLR

**Fig 5:**
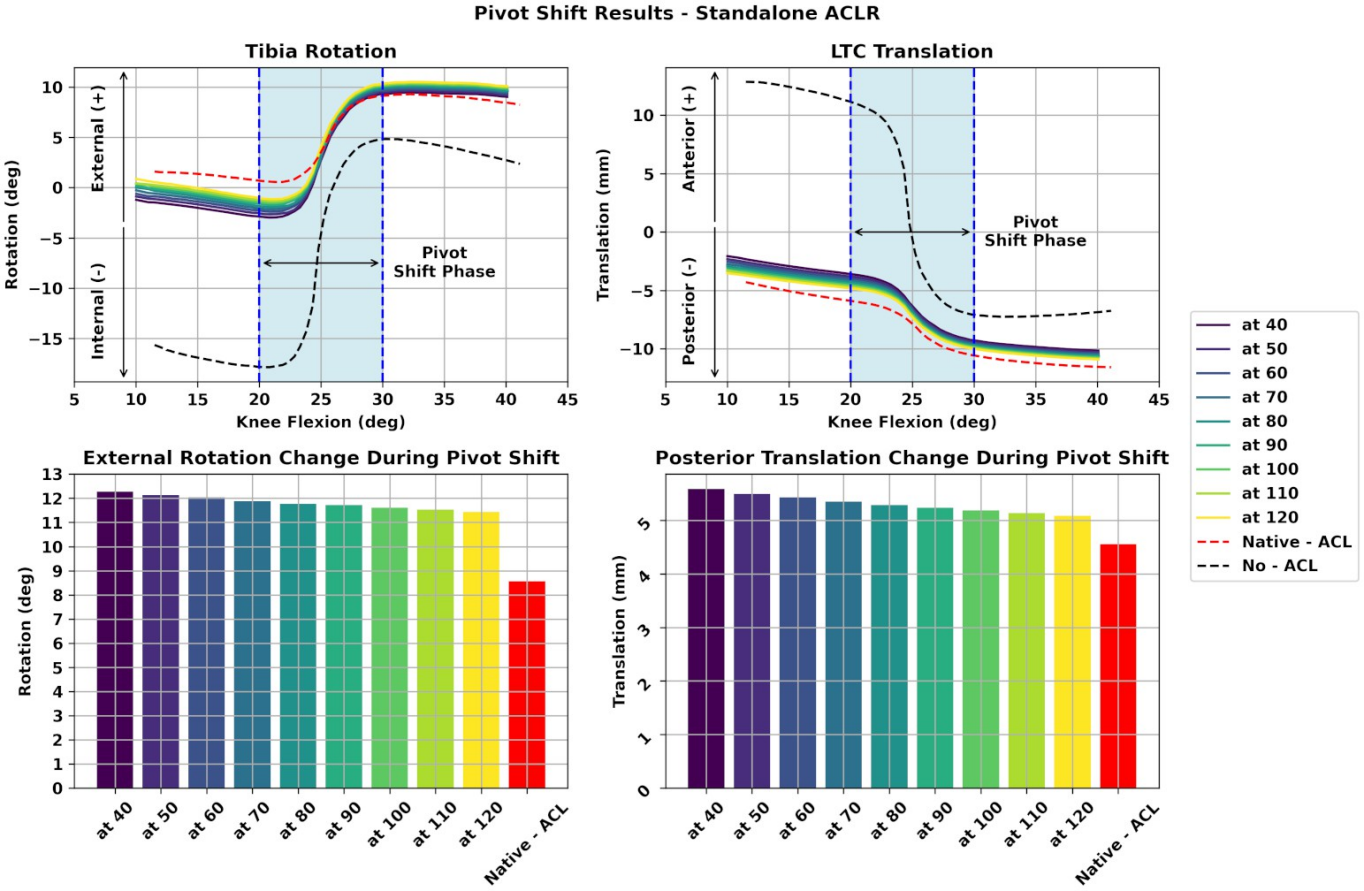
Assessment of standalone ACLR during PS. In this figure, we illustrate the performance of standalone ACLR in restoringITR and ATT. At the bottom we present the magnitude of ETR and PTT ranges during the PS movement using bar plots. As the graft pretension increases, both variables reduce. In both cases, the ACLR knee model exhibits greater ETR and PTT values compared to the Native—ACL model. **(at)**: ACLR graft tension.

Initially, we demonstrate the performance of the standalone SB ACLR surgery during the PS simulation. We varied the ACLR graft pretension in the range of 80 N ±50%. The results are presented in Fig 5. The top charts present the evolution of ETR and PTT per knee flexion angle. At the bottom we provide bar plots that describe the magnitude of each variable based on their range during the PS phase. We observe that increasing the graft tension leads to reduced ETR and PTT. However, the ETR is larger when compared to the Native—ACL model as seen from the bar plot in the lower left part of Fig 5. This holds true for all cases of graft pretension values. We notice the same behavior for the PTT of the LTC where the increase of the graft tension leads to a decrease of PTT and closer to the Native—ACL model. However, we can notice that pretension of the ACLR graft does not have a great effect on these two variables.

### Combined ACLR—LET

**Fig 6:**
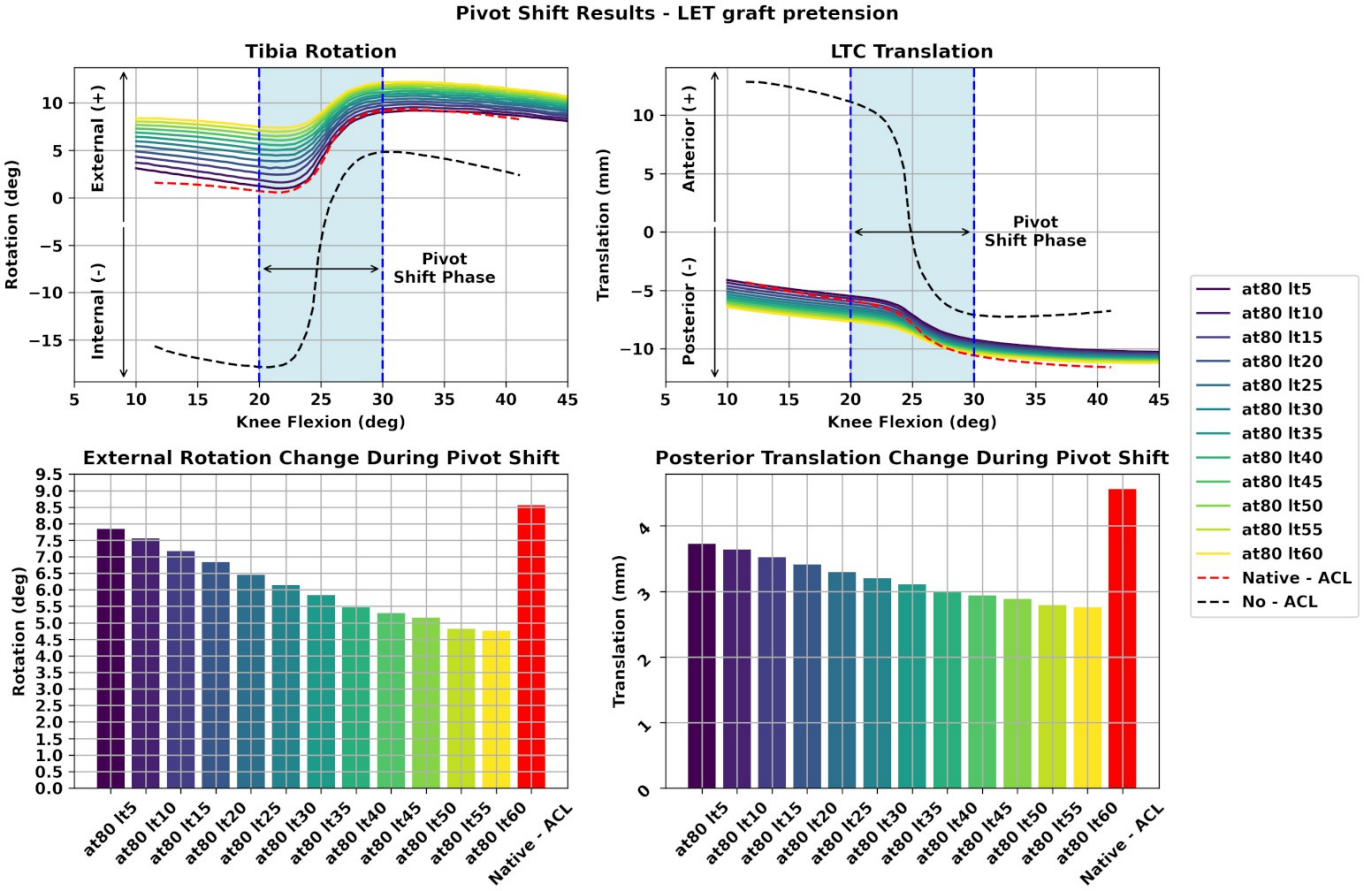
Assessment of LET graft pretension for the combined ACLR—LET during PS. In this figure, we depict the results for the scenario where the LET graft pretension is altered and the ACL graft tension is kept constant. We observe that increasing LET graft pretension leads to decreased ETR and PTT. However, in all cases these values are lower than the performance of the Native—ACL model. **(at)**: ACLR graft tension. **(lt)**: LET graft tension.

Next, we illustrate the performance of the combined ACLR—LET surgery. We modified the LET graft tension while the ACLR graft tension was fixed in a certain value (80 N). The results are depicted in Fig 6. We observe that increasing the graft pretension load leads to reduced ETR and PTT. However, in all cases the achieved ETR is lower than the performance of the Native—ACL model and with a downward trend. The same holds true for the PTT variable as seen from the bar plot on the lower right of Fig 6. Therefore, we can state that increasing the LET graft pretension leads to a decrease in both ETR and PTT. However, larger values of tension may lead to excessive knee constraint for both the ETR and PTT variables.

### Pretension sensitivity analysis for combined ACLR—LET

Subsequently, we decided to identify the relationship between the pretension values for the ACLR and LET grafts, respectively, in the scope of the combined ACLR—LET surgery setup. The objective was to find the optimal combination that leads to reduced but not excessive rotational and translational laxity. Therefore, we analyzed 108 FE models that correspond to all possible combinations of graft tension values for the ACLR and LET grafts. We mention again that the ACL tension values varied in the range of 80 N ±50% with a step of 5 N. The respective LET graft tensions were between 5 N–60 N with a step of 5 N. We decided to depict the results using contour lines for both the ETR and PTT of the LTC. For both variables, our metric was the Mean Absolute Error (MAE) between each combination and the Native—ACL model.

**Fig 7:**
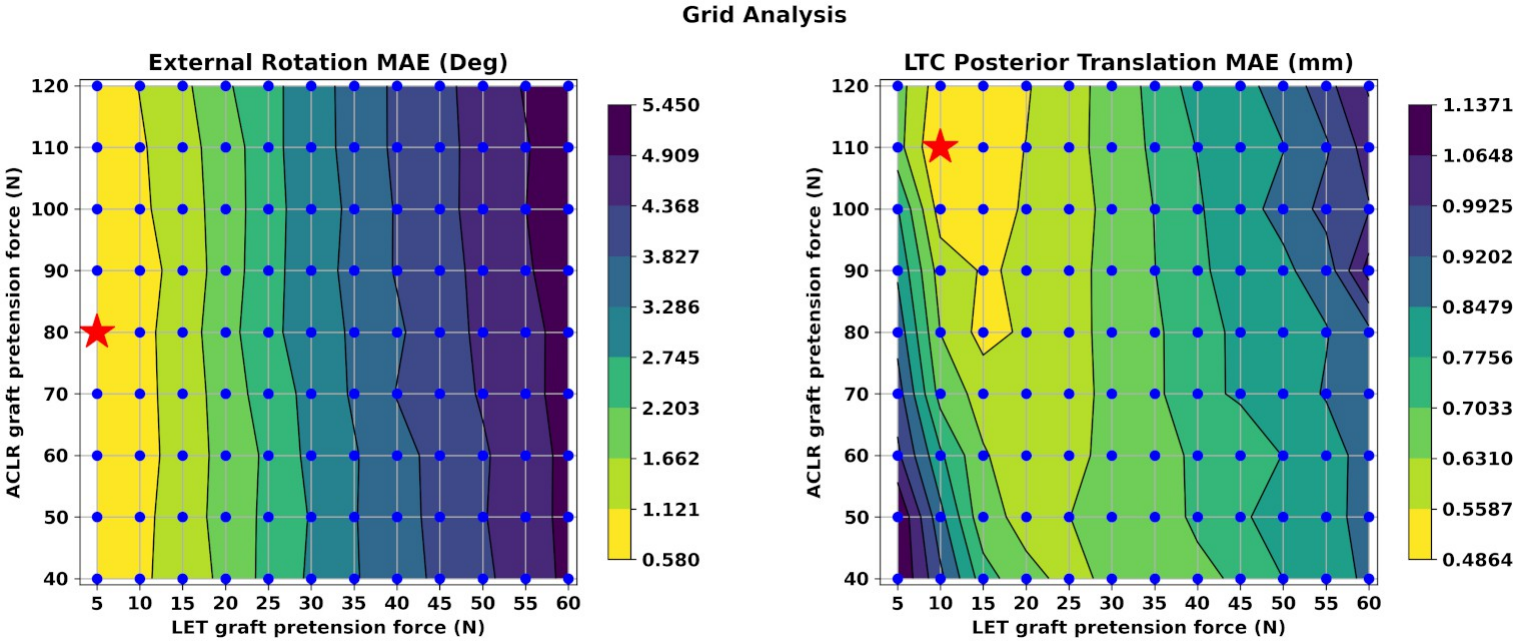
Grid analysis for ACLR and LET graft pretension. In this figure, we present the sensitivity analysis results for all combinations (ACLR tension, LET tension) between ACLR and LET graft pretension values using contour lines. These lines delineate different values of MAE between each combination and the Native—ACL model. The performance of each model is presented by a blue dot. Yellow areas demonstrate the smallest errors whereas purple areas the largest. We also highlight the best combination in terms of minimum MAE with a red star for both variables. These are the pairs (80, 5) and (110, 10) for ETR and PTT, respectively. We observe that ETR is more sensitive to LET tension as illustrated by the almost vertical alignment of the contour plot lines. On the other hand, larger values for both ACLR and LET grafts are required to reduce MAE in PTT.

The contour lines are presented in Fig 7. On the left we present the ETR and on the right the PTT results. Each combination is represented by a blue dot to accentuate its performance. Each colored area contains a range of values for both ETR and PTT that correspond to the MAE of these parameters during the PS movement. With yellow color we denote the lowest values and with purple the largest. Thus, the combinations of ACLR and LET graft tensions that belong to these yellow areas demonstrate the closest performance compared to the healthy knee FE model during the PS reduction phase. Also, we have denoted with a red star the combination featuring the lowest MAE for each variable. In the case of ETR this is the combination of 80 N and 5 N for ACLR and LET graft tension, respectively. The corresponding values for PTT are 110 N and 10 N.

A general remark is that the ETR is more sensitive to the LET graft pretension value as depicted by the almost vertical alignment of the contour lines. Also, the MAE is reduced when we apply a smaller pretension value for the LET graft. On the contrary, we observe that the PTT demonstrates a different, very interesting non—linear behavior. We observe that the combinations with lowest MAE are in the range between 10 N and 20 N of LET graft pretension and from 80 N up to 120 N regarding ACLR graft pretension. Hence, larger values of pretension for both grafts are required to achieve an PTT that is comparable to the Native—ACL model.

### ACLR graft stress comparison

In Table 1, we present the ten best combinations between ACLR and LET graft tensions for the combined ACLR—LET surgery in terms of restoring ITR. The error column contains the MAE in ETR during the reduction PS phase between each reconstructed FE model and the Native—ACL FE model starting from the lowest value. For each combination, we exhibit the corresponding PTT. values. Also, we provide max Von Mises stress values for the ACLR graft around the femoral tunnel insertion site. In an effort to compare the combined method with standalone ACLR surgery we present on the right side of Table 1 the corresponding results for the standalone ACLR surgery.

**Table 1:** The ten best combinations of pretension for the ACLR and LET grafts in the combined ACLR-LET surgery. The term “best” refers to the optimal restoration of native ETR. For each pair, we also provide the corresponding LTC PTT and von Mises stress values around the femoral tunnel.

Combined ACLR—LET				Standalone ACLR			
ACLR-LET Tension (N)	ETR Error (deg) ^†^	LTC PTT Error (mm) ^†^	Stress (MPa) ^‡^	ACLR Tension (N)	ETR Error (deg) ^†^	LTC PTT Error (mm) ^†^	Stress (MPa) ^‡^
80—5	0.659	1.017	21.734	120	1.188	0.781	26.471
60—5	0.691	1.128	20.022	110	1.203	0.876	25.774
70—5	0.701	1.075	21.083	100	1.217	0.973	25.542
110—5	0.707	0.772	23.382	90	1.233	1.067	25.143
90—5	0.717	0.957	22.334	80	1.191	1.156	24.763
120—5	0.745	0.782	23.831	70	1.275	1.299	24.331
100—5	0.763	0.901	22.892	60	1.352	1.436	23.791
40—5	0.821	1.257	18.442	50	1.435	1.575	23.313
90—10	0.826	0.712	21.493	40	1.583	1.742	22.485
50—5	0.846	1.258	19.455	-	-	-	

^†^ The ETR and LTC PTT errors are measured as the MAE during the PS movement between each pair and the Native—ACL model.

^‡^ The von Mises stress corresponds to the maximum Von Mises stress developed in the ACLR graft and around the femoral tunnel insertion area.

We observe that all these combinations include a minimal pretension value of 5 N for the LET graft apart from the (90, 10) combination and feature a ETR MAE less than 1°. On the other hand, the MAE for the ETR is larger in standalone ACLR and for the same ACLR graft pretension values. Also, it increases as the ACLR graft tension decreases. In general, the combined ACLR—LET surgery demonstrates a ETR closer to the Native—ACL FE model compared to the standalone ACLR surgery.

Regarding PTT we notice that in the isolated surgery the MAE increments as the ACLR graft pretension value is reduced. For the combined method, we notice that between the ten combinations of lowest ETR, the pair of 90 N and 10 N of pretension for ACLR and LET grafts demonstrates the lowest MAE for PTT. Also, larger values of ACLR graft pretension lead to the decline of MAE. Hence, we can state that the ITR stability is more affected by the LET graft pretension, whereas larger values of ACLR graft pretension improve ATT stability of the LTC.

Furthermore, we perceive that in all cases the combined approach leads to reduced ACLR graft stresses close to the femoral insertion area and for the same ACLR graft pretension. Normally, the stress is reduced when we apply lower pretension values for the ACLR graft. For example, we notice that for the best combination regarding ETR the ACLR graft pretension is 80 N and the MAE is 0.659° and with a graft stress of 21.734 MPa. For the same pretension the isolated surgery leads to a ETR MAE of 1.188° and a stress of 24.764 MPa. However, in the case of standalone ACLR the minimum MAE is for 120 N of graft pretension that leads to increased graft stress at 26.471 MPa. In general, the differences between graft stresses are moderate. These results highlight that a trade-off between rotational and translational stability and ACLR graft stress should be addressed when planning ACLR surgery.

## Discussion

The essential objective of this study was to assess the effect of the combined ACLR—LET surgery approach on restoring knee kinematics employing a PS FE simulation study. Several clinical studies have been conducted in an effort to investigate whether LET can lead to improved knee rotational stability when performed in addition to ACLR [3, 6, 8]. Also, studies that use *in vivo* navigation and measurement of knee kinematics can be found [9]. However, and to the best of our knowledge, this is the first research work that attempts to shed light in this topic undertaking a FE simulation approach. In this study, we sought to answer the key question of whether a complementary LET operation to the standard ACLR surgery leads to improved stability in terms of ITR and ATT and reduced stress in the ACLR graft. Towards this direction, we modified and deployed an automated modeling workflow that is built upon open-source software tools, and was previously published [18]. FE modeling and simulation is a valuable tool for studying biomechanics. However, FE models are susceptible to modeling assumptions and many FE studies initiate by creating a reference model that usually represents the healthy joint [17, 19]. Similarly, we modified a validated knee model of our previous study [18]. We did not include the patellofemoral joint and the muscles that wrap around the joint. Although these simplifications seem to eliminate essential details of the physical counterpart, we can claim that our model representation is good enough for the scope of this study. We are not experimenting with dynamic voluntary movements, such as gait. Rather, we assess the surgery models by simulating a passive clinical exam, the PS. Moreover, similar choices can be found in FE literature [16, 19, 22, 32]. Regarding knee joint kinematics, we adopted tibia and femur reference frames that were optimized using experimental data from the OpenKnee (s) project.

The development of the validated FE model provided us a baseline to evaluate ensuing FE simulations and perform “what—if” scenarios with a strong confidence in our results. Apart from a validated model that is a surrogate representation of the physical counterpart we had to also apply realistic boundary conditions. Thus, the first step was to design a loading profile that closely resembles the forces and torques applied to the knee joint during the real world PS clinical exam. A complete description and discussion of the adopted PS profile is provided in S1 File. After assessing the performance of the reference model with an effective PS, we proceeded with the assembly of FE models that represented the ACLR knee joint. We validated these models in terms of material properties using experimental data from uniaxial tests [34, 42]. Moreover, we aimed to provide results for von Mises stress around the femoral tunnel insertion region, similar to other FE studies [17, 19, 21, 22, 43]. Thus, we performed mesh density tests that were crucial to enhance the fidelity of our simulation results. Subsequently, we used these validated models in FE simulations of the PS clinical exam to answer the key questions of our study. We assessed the effect of graft pretension in restraining ETR and PTT during PS for both ACLR and LET. We used the range of each variable during the PS reduction phase as a measurement. Regarding ETR, we observed that the standalone SB ACLR surgery exhibits a reduced ETR as the graft tension value increases. However, this ETR is larger than the Native—ACL model regardless of graft pretension with values ranging from 2.96° up to approximately 3.5°. The same remarks are evident when observing the graphs for PTT of the LTC. However, in these cases the absolute difference between the ACLR models and the Native—ACL are smaller than in ETR. These results imply that the standalone ACLR surgery could be susceptible to increased rotatory instability and PTT. Also, a larger value of graft tension is required to reduce ETR and PTT and align them with the Native—ACL model.

We compared these findings with the combined ACLR—LET technique. Initially, we assigned different values of graft pretension to the LET graft while the ACLR graft tension was kept fixed to a common literature value. We estimated again the range of ETR and PTT during the PS phase. We observed that the combined ACLR—LET exhibits lower values regarding both ETR and PTT compared to the isolated method as illustrated in Fig 6. The difference is glaring in the rotational stability where we observe values ranging from 4.481° up to 8.002° for the ETR. The baseline measurement was about 8.5°. Moreover, we noticed that increasing the LET graft pretension leads to excessive decrease of the ETR and PTT. In fact, a level of ETR that is half the performance of the Native—ACL model is evident for the larger values of LET graft pretension.

As a general verdict we can state that the combined ACLR—LET leads to reduced PTT and ETR compared to the healthy reference model. This conclusion is similar to relevant clinical studies [4, 6, 44–46] where PS tests were used to assess knee kinematics restoration either by performing cadaver experiments or systematic reviews of clinical trials and studies on the same field. Even though these primary outcomes were quite promising, we assumed that a more comprehensive analysis would shed additional light on this domain. Thus, we attempted to identify which combination between the pretension values assigned to the grafts in the case of ACLR—LET surgery leads to knee kinematics that are similar to the Native—ACL model. This time, we adopted MAE as our metric. The almost vertical lines of the contour plots presented in Fig 7 showcase the sensitivity of the combined method to the LET graft pretension regarding ETR. Moreover, the best results appear on the far left of the contour and in regions were the FE models with minimal LET tension values of 5 N and 10 N lie. In fact, the best combination is the pair of 80 N and 5 N of ACLR and LET tension, respectively. These results reveal that a minimal LET graft tension should be used to avoid over constraining the knee in terms of ETR. This result is similar to the methodology of clinical papers where minimal values of pretension in LET are capable of limiting the PS phenomenon without excessive constraint of the knee rotation [3, 47]. On the other hand, large pretension values lead to an overconstrained knee behavior regarding ETR [28]. However, while knee overconstraint has been reported in similar clinical studies, its effect on knee range of motion and early OA onset is still a debatable topic [48–51].

On the contrary, we notice a different behavior regarding PTT, as depicted on the right side of Fig 7. In this case, the combinations with ACLR graft tension ranging from 80 N–120 N and LET pretension in the range of 10 N–20 N exhibit the lowest MAE. The most efficient combination is the 120 N and 10 N for the ACLR and LET pretension, respectively. Hence, we notice that increased reduction of PTT requires a higher pretension force for both grafts to be comparable to the performance of the Native—ACL model. Furthermore, we notice that the combined method leads to lower PTT MAE compared to the standalone ACLR surgery and for the same ACLR graft pretension. However, these discrepancies are moderate in most cases. Similar results are reported in clinical studies [6, 52]. Nonetheless, graft pretension is a debated issue among clinicians as it is clear from the large variance between applied forces [8, 28, 46, 47]. Our findings suggest minimal graft pretension level, although subject specific characteristics, and the inherent assumptions of the simulation approach should not be neglected. We should also state that the graft tension during surgery can potentially decrease over time due to viscoelastic effects and bone reaction forces after fixation. Thus, any suggestion about precise graft pretension values should be made in caution.

Additionally, a potential advantage of performing LET is the reduced stress developed in the ACLR graft leading to lower graft failure chances. We presented max von Mises stress for the ACLR graft elements around the femoral tunnel insertion area. We observed that for identical pretension values the combined method leads to a modest decrease in graft stress with a mean value of 2.93 Mpa. The decrease in graft stress seems to be larger for lower values of graft tension. The biggest decrease is 4.04 MPa and corresponds to the 40 N of ACLR graft pretension. Graft stress reduction is reported also by similar clinical studies [6, 53]. LET appears to act in a protective way regarding the ACLR graft. Nonetheless, the grade at which the ACLR graft is offloaded is still unclear and depends on several parameters, such as graft fixation angle and graft pretension. Also, the choice of passing the LET graft under or superficially to LCL seems to affect ACLR graft stress reduction [3, 6]. Still, this stress reduction is a potential contributor to the reduced graft failure rates when a combined surgery is adopted [3, 6, 53].

Although these results are promising and in general agreement with relevant clinical research, our study does not come without limitations that are inherent in FE modeling and simulation. These include loading boundary conditions, contact models, and material properties. We tried to validate each step of the modeling workflow using experimental data. Moreover, our modeling assumptions are justifiable in the case of a passive clinical exam such as the PS. However, the ACL injuries are most common in demanding physical tasks. We anticipate that all these limitations can be interesting directions of future work that could further enlighten the different aspects of ACLR. We would like to assess the impact of LET in FE simulations that involve boundary conditions from rigid multibody dynamics resembling such activities. For example, the developed lateral tibiofemoral contact pressures after LET or the impact of ITB debilitation to knee loading due to graft harvesting are debatable topics that we could address [28, 37]. The same holds true for surgical choices such as femoral graft fixation using overlay anchor fixation instead of femoral tunnel drilling, and the LET graft passage beneath or over the LCL [54]. Moreover, in the scope of this work we developed a loading profile that seems to trigger the desired PS movement for the given knee model. We should apply the profile in knee FE models from multiple subjects to obtain a more systematic assessment of the loading conditions and the applied surgery methods. Also, we wish to expand our analysis to investigate additional ACLR surgery techniques, such as the Anterolateral Ligament Reconstruction (ALLR). Nonetheless, the results of this study showcase the potential of our FE modeling workflow. Thus, we could materialize these prospective research objectives by improving the already developed modeling tools.

## Conclusion

In this study, the main focus was the FE modeling and PS simulation of the combined ACLR—LET surgery in an effort to investigate its advantages and implications. The FE model assembly process is materialized and automated through a custom Python module. This module creates FE models that can be solved by the FEBio solver. We made this module publicly available [55]. We provide all models included in this study to the research community along with scripts that reproduce our results to solidify research pellucidity. The FE models can be used and modified from fellow researchers to explore a variety of ACLR topics and provide feedback and improvements in our work. In conclusion, we envisage that the tools developed for the purpose of this study supported by the promising relevant clinical results can emerge to a valuable supporting framework available to clinicians and biomedical researchers for ACLR study in a pre—surgery planning setting.

## Supporting information

S1 File Supporting information related to this publication. (PDF)
