# Supplementary material for: Can lateral tenodesis improve the rotational stability of the ACL reconstruction? A finite element analysis
Source: PLoS One. 2024 Feb 27;19(2):e0293161. doi: 10.1371/journal.pone.0293161 (PMC10898738; doi:10.1371/journal.pone.0293161)
Supplement: S1 File — (PDF) [file pone.0293161.s001.pdf]

Konstantinos Risvas[1,*], Dimitar Stanev[1,2], and Konstantinos Moustakas[1]

[1]Department of Electrical and Computer Engineering, University of Patras, Patras, Greece

[2]École Polytechnique Fédérale de Lausanne, Institute of Bioengineering, Lausanne, Switzerland

[*]krisvas@ece.upatras.gr

Supplementary Material

1 Introduction

In this document, we provide additional information about the adopted methodology presented in the main manuscript. First, we will present the mathematical formulae for the graft constitutive material models. We used these to fit experimental data and assign realistic material properties in our Finite Element (FE) models. We will also demonstrate the results of mesh density and Bulk modulus sensitivity tests. Subsequently we will provide the results for the Native - Anterior Cruciate Ligament (ACL) model validation and an assessment of the adopted Pivot - Shift (PS) profile. Moreover, we will provide the methodology for applying graft pretension using Spherical Linear Interpolation (SLERP). Additionally, we will describe the methodology of estimating the projections of Tibia Medial Compartment (TMC) and Tibia Lateral Compartment (TLC) on the femoral mesh surface. Finally, we will provide a brief context of the adopted steps for post - processing the FE simulation results.

2 Material Validation Tests

In this section, we provide additional information regarding fine-tuning of the material properties assigned to the graft meshes. We will derive the equations that we are going to use for polynomial fitting of the material properties in experimental data of uniaxial tests.

2.1 Experimental Data Fitting

The grafts were modeled with an appropriate hyperelastic transversely isotropic Mooney - Rivlin material featuring an uncoupled deviatoric and volumetric behavior [22, 15]. Thus, the deformation gradient can be decomposed as follows: $\mathbf{F} = J^{1/3}\tilde{\mathbf{F}}$, where $\tilde{\mathbf{F}} = J^{-1/3}$ is the modified deformation gradient, characterizing a volume-preserving (or isochoric or deviatoric) deformation and J being the Jacobian of the deformation. On the other hand, the term $J^{1/3}$ corresponds to the volume-changing (or dilatational) component of deformation. It holds that $\det(\mathbf{F}) = \det(J^{1/3}) = J$. Consequently, the right Cauchy - Green strain tensor can be modified as follows:

$$\mathbf{C} = \mathbf{F^T F} = (J^{1/3}\tilde{\mathbf{F}})^{\mathbf{T}} J^{1/3}\tilde{\mathbf{F}} = \tilde{\mathbf{F}}^{\mathbf{T}} J^{2/3}\tilde{\mathbf{F}} = J^{2/3}\tilde{\mathbf{F}}^{\mathbf{T}}\tilde{\mathbf{F}} = J^{2/3}\tilde{\mathbf{C}} \tag{1}$$

Here, $\tilde{\mathbf{C}}$ is the modified right Cauchy-Green deformation tensor. Following that, the adopted formulation for the scalar-valued uncoupled density strain energy function undertakes the following form [22]:

$$\begin{aligned} \Psi(\tilde{\mathbf{C}}) &= \Psi_{iso}(\tilde{\mathbf{C}}) + \Psi_{vol}(J) \\ &= \underbrace{F_1(\tilde{I_1}, \tilde{I_2}) + F_2(\tilde{\lambda})}_{\Psi_{iso}} + \frac{K}{2}[\ln(J)]^2 \end{aligned} \tag{2}$$

Here, $\tilde{I_1} = \mathrm{tr}(\tilde{\mathbf{C}}) = \tilde{\lambda_1}^2 + \tilde{\lambda_2}^2 + \tilde{\lambda_3}^2$ and $\tilde{I_2} = 1/2[(\mathrm{tr}(\tilde{\mathbf{C}}))^2 - \mathrm{tr}(\tilde{\mathbf{C}}^{\mathbf{2}})]$ are the first and second invariants of $\tilde{\mathbf{C}}$, respectively. Also, $\tilde{\lambda}$ is the modified (deviatoric) stretch, with $\tilde{\lambda} = J^{-1/3}\lambda$, and $\tilde{\lambda}^2 = \mathbf{a_0}\tilde{\mathbf{C}}\mathbf{a_0}$, where $\mathbf{a_0}$ is the material fiber direction in the reference configuration.

Regarding the different components of Equation 2, the function F_1 represents the isotropic behavior of the ground substance matrix and is defined as $F_1(\tilde{I_1}, \tilde{I_2}) = C_1(\tilde{I_1} - 3) + C_2(\tilde{I_2} - 3)$. On the other hand function F_2 describes the tensile-only behavior of the collagen fibers and is defined as follows:

$$F_2(\tilde{\lambda}) = \begin{cases} 0 & \text{if } \tilde{\lambda} < 1 \\ C_3(e^{-C_4}(\mathrm{Ei}(C_4\tilde{\lambda}) - \mathrm{Ei}(C_4)) - \ln\tilde{\lambda}) & \text{if } 1 < \lambda < \lambda^* \\ C_5(\tilde{\lambda} - 1) + C_6 \ln\tilde{\lambda} & \text{if } \tilde{\lambda} \geq \lambda^* \end{cases} \tag{3}$$

where,

$$C_6 = C_3(\exp(C_4(\lambda^* - 1)) - 1) - C_5\lambda^* \tag{4}$$

In Equation 3 C_1, and C_1 are the material coefficients for the Mooney-Rivlin material. When $C_2 = 0$, then the model reduces to the uncoupled Neo-Hookean model. Respectively, in Equation 4, $\tilde{\lambda}$ is the fiber stretch along the fiber direction, λ^* is the transition stretch value where the material collagen fibers start to straighten, C_3 scales the exponential stress, C_4 is the rate at which the fibers uncrimp, and C_5, is straightened collagen modulus. C_6 is estimated based on the condition that the estimated stress is continuous at λ^*. All the aforementioned material coefficients are determined by curve-fitting to experimental measures of stress - strain relationship for the material of interest. Towards this objective, the strain energy function needs to be manipulated to a form capable of estimating fiber stress.

For F_1 we can estimate the principal stresses of the Cauchy stress by taking the derivative of the strain energy function with respect to the invariants and multiplying with the corresponding stretch. Thus,

$$\sigma_1 = \tilde{\lambda}_1 \frac{\partial F_1}{\partial \tilde{\lambda}_1} = \tilde{\lambda}_1 \frac{\partial F_1}{\partial \tilde{I}_1} \frac{\partial \tilde{I}_1}{\partial \tilde{\lambda}_1} = 2C1\tilde{\lambda}_1^{\ 2} \tag{5}$$

$$\sigma_3 = \tilde{\lambda}_3 \frac{\partial F_2}{\partial \tilde{\lambda}_3} = \tilde{\lambda}_3 \frac{\partial F_1}{\partial \tilde{I}_1} \frac{\partial \tilde{I}_1}{\partial \tilde{\lambda}_3} = 2C1\tilde{\lambda}_3^{\ 2} \tag{6}$$

However, for a uniaxial test and assuming incompressibility we have $\tilde{\lambda} = \tilde{\lambda}_1$ and $\tilde{\lambda}_2 = \tilde{\lambda}_3 = 1/\sqrt{\tilde{\lambda}}$. Also, $\sigma_3 = 0$. Substituting Equation 6 from Equation 5 we have:

$$\begin{aligned} \sigma_1 - \sigma_3 &= 2C1\tilde{\lambda}_1^{\ 2} - 2C1\tilde{\lambda}_3^{\ 2} \Rightarrow \\ \sigma_1 &= 2C1(\tilde{\lambda}^2 - 1/\tilde{\lambda}) \end{aligned} \tag{7}$$

Regarding F_2, the fiber stress can be related to its derivative with respect to $\tilde{\lambda}$ as follows:

$$\tilde{\lambda} \frac{\partial F_2}{\partial \tilde{\lambda}} = \begin{cases} 0 & \text{if } \tilde{\lambda} < 1 \\ C_3(e^{C_4(\tilde{\lambda}-1)} - 1) & \text{if } 1 < \lambda < \lambda^* \\ C_5\tilde{\lambda} + C_6 & \text{if } \tilde{\lambda} \geq \lambda^* \end{cases} \tag{8}$$

Depending on the experimental data it is common to divide the principal stress by the stretch $\tilde{\lambda}$ to acquire the engineering stress and perform curve-fitting. Regarding the dilational part of the strain energy function, the term K is the bulk modulus, which in our study was selected as a multiple of the C_1 coefficient of Equation 8 in the range of $100 < (K/C_2) < 10000$ to achieve nearly incompressible behavior. Experimental data from uniaxial tests for the quadruple semitendinosus tendon and iliotibial band tissues were used to fit the polynomials of Equation 7 and Equation 8 [7, 6]. These tissues are commonly used among orthopedic surgeons during Anterior Cruciate Ligament Reconstruction (ACLR) and Lateral Extra-Articular Tenodesis (LET) [16, 4, 10]. The fitted curve and the estimated transition point are presented in Fig S1 .

Also, the adopted material model requires the definition of a single fiber direction, either for the entire mesh or for each individual element. In our study, we defined a local fiber direction for each element that follows the long axis direction of the entire graft mesh.

2.2 Mesh Density

Next, we present the mesh convergence studies for each graft material. The convergence measure was set to the maximum Von Mises stress. We defined an error tolerance of 5% from the converged value for a nominal strain of 10%. The results are presented in Fig S2. For the quadruple semitendinosus graft the converged value was 17.0 MPa for

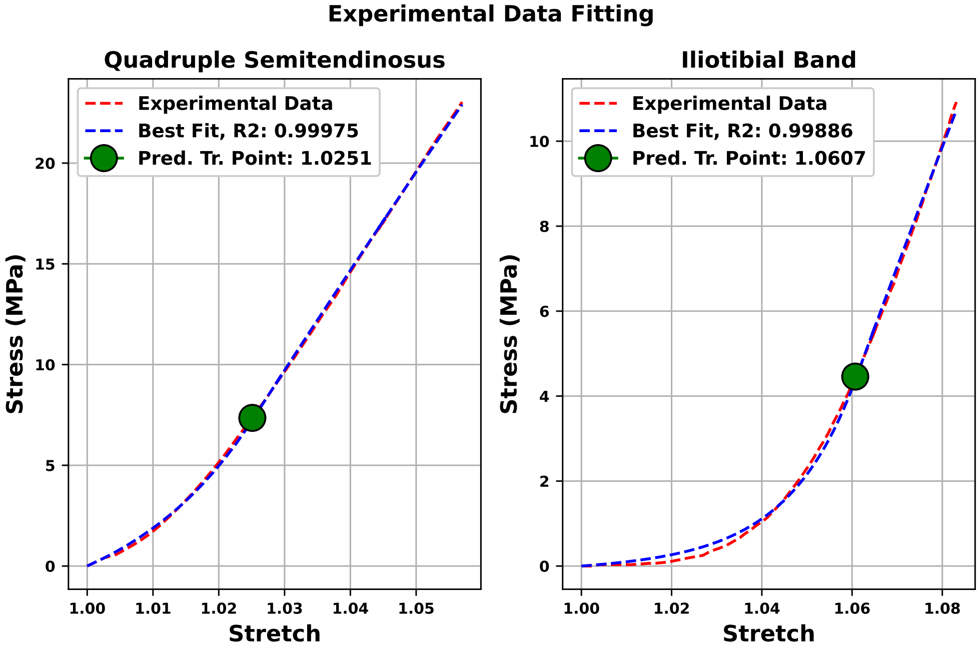

S1 Figure. Curve - fitting for material properties. We fitted the material polynomials in experimental stress - strain curves to obtain realistic material properties. The curves correspond to uniaxial material tests [17, 7].

a total of 23040 elements (Fig. S2). Based on the adopted tolerance we considered an optimal number of 15360 elements. On the other hand, for the iliotibial band material we found an optimal number of elements of 12928 for a converged value of 125.162 MPa.

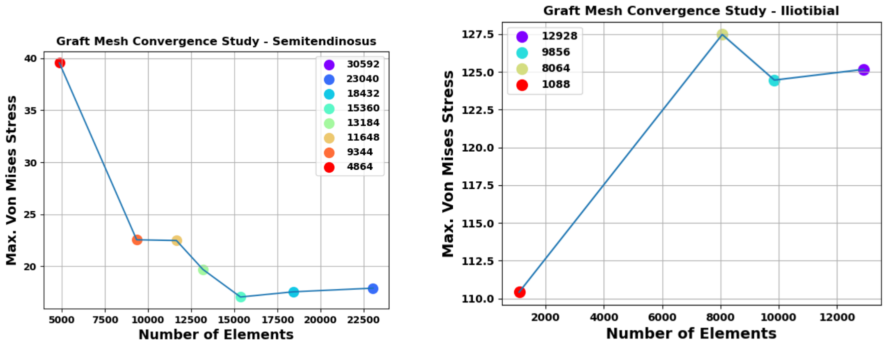

(a) **(b)**

S2 Figure. Mesh density tests for graft materials. a) Semitendinosus graft, b) Iliotibial Band graft.

2.3 Bulk Modulus

After determining the optimal mesh density, we used the respective graft meshes in uniaxial simulation studies to determine the optimal value for the Bulk modulus [1]. We varied the Bulk modulus value in the range of $100*C1 < K < 10000*C1$, with $C1$ being the first coefficient of the adopted material model. $C1$ was adopted from literature, since we did not have access to experimental data of biaxial tests [14]. A deformation of 10% was applied to the graft mesh. We utilized the "straight" graft meshes and the nodes corresponding to the bone fixation parts were attached to two rigid bodies (physical reference frames), located at the graft ends. Then, a displacement was applied to one of the rigid bodies to enforce the desired uniaxial deformation while the other rigid body was held fixed across all Degrees Of Freedom (DoFs). At the end of each simulation, we measured the 'Volume Ratio' with values. Suitable values are very close to 1 signaling a nearly incompressible behavior. We present indicative results in Table S1. A Bulk modulus of 10000 seems to cause a volume ratio close to 1.

Bulk's Modulus	Volume Ratio
100	1.1011
1000	1.022
2000	1.011
3000	1.008
4000	1.006
5000	1.0049
6000	1.0039
7000	1.0037
8000	1.0035
9000	1.0032
10000	1.0031

S1 Table. Bulk Modulus Sensitivity Analysis Results. We observe that incompresibility is achieved for a Bulk's modulus of 10000.

The final adopted values for the two grafts are presented in Table S2.

Parameter	ACLR Graft	LET Graft
**c1**	2.75†	2.75†
**c2**	0†	0†
**c3**	0.065†	0.065†
**c4**	51.63†	112.0971
**c5**	549.48	490.668
**l_max**	1.0251	1.0182
**k**	10000	10000
**fiber**	[1,2]	[1,2]

† Values were acquired from literature [14, 13].

S2 Table. Graft material properties.

Native - ACL model validation

In Fig S3, we present the sensitivity analysis results for the pre-strain assigned to the ACL material model. The experimental reference line is represented with red color. The optimal fiber stretch in our study was found to be a value of 1.06. For this value, the mean squared error (MSE) between the simulated Anterior Tibial Translation (ATT) and the reference red line is the lowest.

PS profile assessment

Subsequently, we discuss the effectiveness of the adopted PS profile, which is depicted in Fig 3 of the main manuscript. We applied the loading profile to the FE models representing the injured and healthy knee joint respectively and evaluated the Posterior Tibial Translation (PTT) and External Tibial Rotation (ETR). The results are presented in Fig S4. We observe that the PS characteristic features are evident as depicted by the abrupt changes in both ETR and PTT. The PS movement occurs at a knee flexion angle of approximately 25°. The applied loads cause the tibia in the ACL deficient knee (black dashed line) to start from an initially subluxed configuration that is characterized by the excessive ATT of the TLC and an increased Internal Tibial Rotation (ITR). The tibia is rapidly rotated externally and translated posteriorly highlighting the two prominent features of the clinical PS starting at approximately 23° of knee flexion angle. The ETR magnitude during the reduction phase is 19.94° and the respective PTT is 16.89 mm. We also observe similar behavior for the Native - ACL. However the magnitude for both variables is much lower with an ETR of 8.159° and an PTT of 4.656 mm.

In similar FE ACLR studies, the authors adopted a "subluxation" PS profile that included internal tibial and valgus torques. The knee flexion was fixed in certain angles

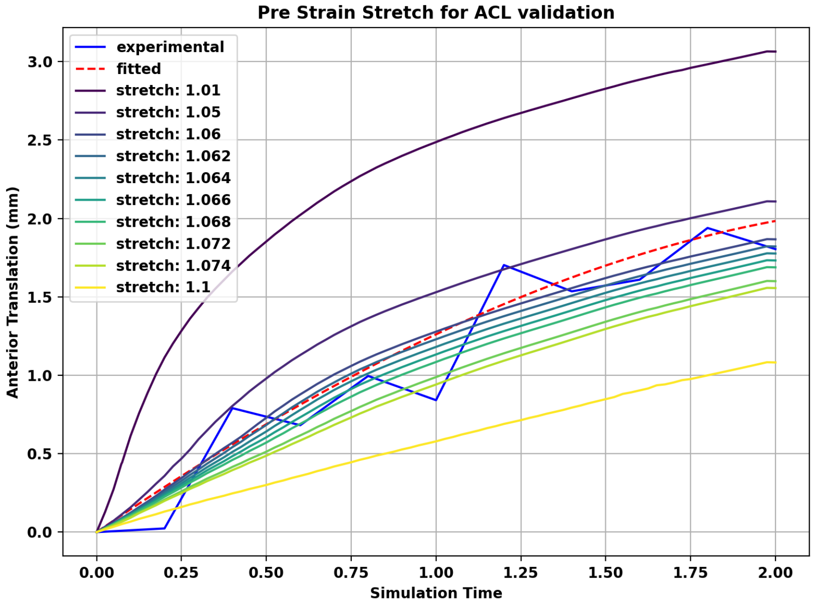

S3 Figure. Validation of Native - ACL model regarding ACL pre - strain. In this figure, we present the results for identifying the optimal fiber stretch that was applied as *in situ* strain in the ACL material. Since this strain affects joint mechanics, we performed a series fo FE simulations to reproduce an anterior drawer experiment from the OpenKnee (s) project. During each simulation we altered the fiber stretch appointed to the ACL material, applied the experimental loads and measured ATT. We estimated the MSE between simulated ATT and the red reference line which was fitted to the experimental ATT (blue line). The lowest MSE was found for a fiber stretch of 1.06.

(0°, 30° [20, 21, 8]. The values for internal rotation moment ranged from 4Nm up to 7.5Nm, whereas the valgus torques ranged from 6.9Nm up to 10Nm [20, 21]. Anterior tibia forces of 103N or 134N were also included during the subluxation phase [20, 8, 21]. In other FE studies the PS was a combination of a knee flexion of 20° and a tibia torsion about a vertical axis [19, 18]. Although, our PS profile acts on the reverse direction compared to the mentioned FE studies, the adopted values for the torques are similar. Additionally, in our study the knee is set free during the flexion phase without applying the PS test in a fixed angle in contrast with other similar FE studies.

Comparing our results with clinical studies that performed PS tests using robot simulators, we can find values in the range of (0.5, 5.0) mm for PTT and $-0.01°$ - 3.0° for ETR of the Native - ACL knee [2]. In the same study, a clinically performed PS demonstrated a PTT of 10 mm and an ETR of approximately 15°. Loading profiles from this study were also adopted by other clinical trials [9]. Other studies reported values of 8 up to 12 mm of PTT and 15° - 21° of tibia rotation [12]. Similarly, several studies were performed where a "subluxation" PS test was applied. A simulated PS in a clinical

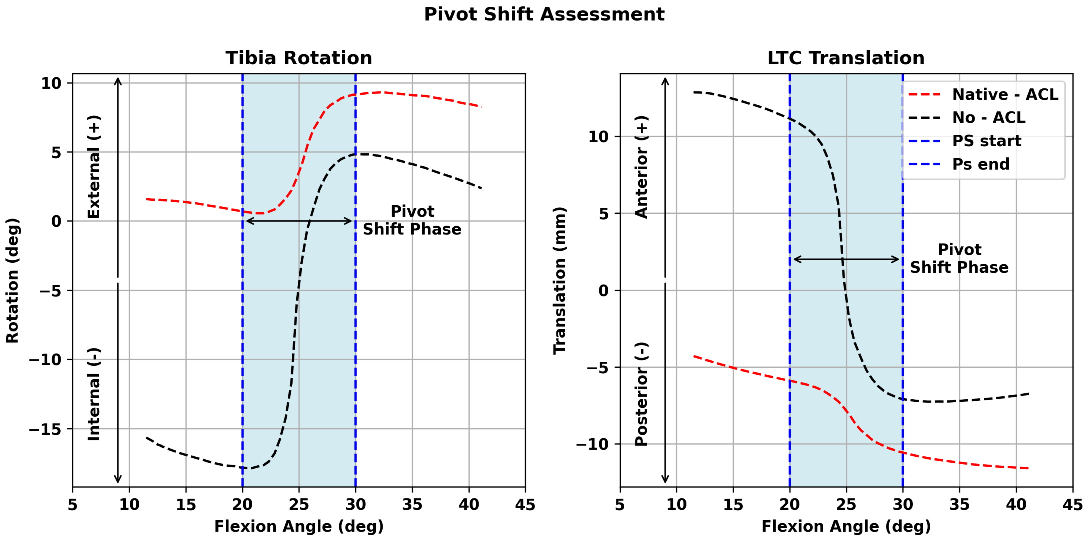

S4 Figure. PS loading profile assessment. In this figure, we illustrate the effect of the applied PS loading profile on the ITR and ATT for both the No - ACL and Native - ACL cases, respectively. The PS movement is clearly evident at about 25° of knee flexion with an abrupt ETR and PTT, respectively. The PS phase is highlighted with light blue color and spans the range between 20° - 30°. The adopted PS is qualitatively similar to clinical and simulated PS profiles [2, 12]. (LC): TLC, (PS): Pivot Shift.

setting showcased an anterolateral subluxation of 12.5 mm at a flexion angle of 30° [3]. In an other clinical study, 25 examiners performed a PS test on two cadaver knees with different degree of rotational laxity [11]. The results for the deficient knee demonstrated an average PTT of 8.8 mm in the low - laxity knee and an average of 17.3 mm in the high - laxity joint. The respective values for ETR were 10.0° and 19.3°. The values for the high - laxity knee are almost identical with our findings. The disparity in PTT can be associated with the difference in the applied anterior force during the PS. However, we selected a force of 25N, which is close to the typical femur weight when the clinician raises the leg before applying the PS test [12]. It is also close to the 30N of other clinical LET studies that apply a simulated PS test [10, 2]. Likewise, discrepancies in ETR can be related to differences in external torque applied to the tibia. Nonetheless, the PS profile exhibits similar qualitative behavior and comparable values for both PTT and ETR. Therefore, we can claim that the adopted profile is capable of producing a PS simulation setup to assess the performance of ACLR surgery techniques.

3 Graft Pretension

In this section, we will provide the mathematical formulation for applying the LET graft pretension. We start by defining the orientation of the tunnel where the graft is going to

be pulled through. Towards this direction, we use the landmarks that define the surgery planning trajectory inside the respective bone (Fig S5).

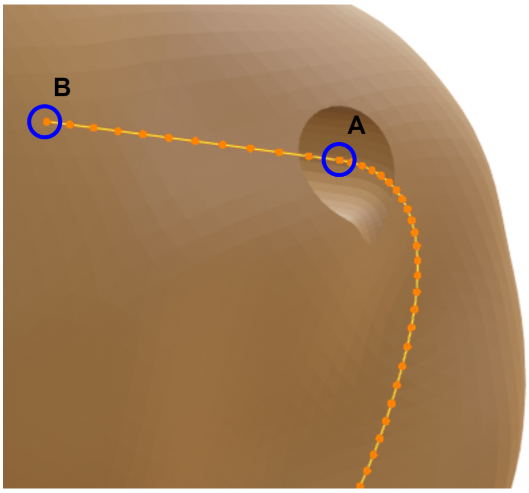

S5 Figure. Osseous tunnel orientation. We use the curve part inside the osseous tunnel to specify the tunnel orientation. This is the normalized vector between points A and B.

Thus, if we define as $\mathbf{d}$ the vector between the points A and B, the direction of this vector is given by the unit vector:
$\hat{\mathbf{d}} = \mathbf{d}/||\mathbf{d}||^2$

Then, we performed a forward FE simulation where we prescribe a knee flexion angle in the range of 0° - 90° with a step of 10°. At the end of the simulation, we extracted the position and orientation of the femoral reference frame. Specifically, we obtained the origin's position vector and the unit quaternion for each step.

A unit quaternion is an alternative mathematical notation for representing the rotation about a rotation axis with direction $\hat{\mathbf{e}}$ and for an angle θ. It has many advantages over the traditional rotation matrices. First of all, it requires only 4 parameters compared to the 9 parameters of the $3x3$ rotation matrix. Additionally, quaternions are free of singularities that are inherent to other rotation representation approaches, such as Euler angles. Finally, performing successive rotations in computer calculations may lead to drifts due to numerical errors. A quaternion can be normalized after each step to represent a rotation. On the other hand rotation matrices tend to loose their orthogonality after each step and these errors are difficult to track and resolve. Hence, quaternions can be easily interpolated.

For an axis with direction vector $\hat{\mathbf{e}} = e_x\mathbf{i} + e_y\mathbf{j} + e_z\mathbf{k}$ and an angle of rotation θ, the mathematical formula for the corresponding unit quaternion is:

$$\mathbf{q} = \cos(\theta/2) + \sin(\theta/2)(e_x\mathbf{i} + e_y\mathbf{j} + e_z\mathbf{k}) \tag{9}$$

In Equation 9, $\cos(\theta/2)$ is the scalar part and $\sin(\theta/2)(e_x\mathbf{i}+e_y\mathbf{j}+e_z\mathbf{k})$ is the vector part.

To rotate an ordinary vector $\mathbf{b} = b_x\mathbf{i}+b_y\mathbf{j}+b_z\mathbf{k}$ we create a quaternion $\mathbf{b_q}$ where the vector part is the vector $\mathbf{b}$. Then, we estimate the conjugation of $\mathbf{b_q}$ by the unit quaternion $\mathbf{q}$.

$$\mathbf{b'_q} = \mathbf{q}\mathbf{b_q}\mathbf{q^{-1}} \tag{10}$$

The quaternion $\mathbf{q^{-1}} = \cos(\theta/2) - \sin(\theta/2)(e_x\mathbf{i}+e_y\mathbf{j}+e_z\mathbf{k})$ is the conjugate of $\mathbf{q}$.

The rotated vector corresponds to the vector part of the quaternion $\mathbf{b'_q}$. Moreover, for n successive rotations we define $\mathbf{q'} = \mathbf{q_n}\mathbf{q_{n-1}}...\mathbf{q_2}\mathbf{q_1}$ where the rotations occur in the reverse order of the multiplication order.

As we mentioned, one advantage of quaternions is that they can be easily interpolated. A common interpolation formulation is the SLERP approach and is described as follows [23]:

$$\mathrm{Slerp}(\mathbf{q1},\mathbf{q2};t) = (\mathbf{q_2}\mathbf{q_1}^{-1})^t\mathbf{q_1} \tag{11}$$

In the above formula $0 \leq t \leq 1$. Hence, we can specify a vector of values for t starting from 0, up to 1 and apply the above formula to create interpolated quaternions between $\mathbf{q_1}$ and $\mathbf{q_2}$.

In our case, we used SLERP to interpolate the quaternion that we acquired from the forward simulation and describes the rotation of the femoral reference frame. In FEBio the first quaternion extracted after a simulation corresponds to a the identity quaternion $\mathbf{q0} = 1+0\mathbf{i}+0\mathbf{j}+0\mathbf{k}$. The last quaternion corresponds to a knee flexion of 90°. Using SLERP, we can estimate the orientation of the reference frame at any given knee flexion angle. For example, if we define $t = [0, 0.3, 0.6, 0.9]$ and apply $\mathrm{Slerp}(\mathbf{q_1},\mathbf{q_2} : t)$ where $\mathbf{q_1} = \mathbf{q_0}$ and $\mathbf{q_2}$ is the quaternion extracted from FEBio for a knee flexion angle of 90° then we can have the femoral frame orientation at 0°, 30°, 60°, and 90°.

To acquire the direction of the osseous tunnel at the desired fixation angle we first create a quaternion $\mathbf{b_q}$ with the vector $\mathbf{b}$ as its vector part. Then, we find the new rotated direction vector by extraction of the vector part of the following conjugation with the desired quaternion $\mathbf{q}$.

$$\mathbf{b_q}' = \mathbf{q}\mathbf{d_q}\mathbf{q}^{-1} \tag{12}$$

Finally, we can apply the pretension force with magnitude F by specifying the following force vector:

$$\mathbf{F} = F\mathbf{d}' \tag{13}$$

This force vector is defined in FEBio as a surface load and is applied to the corresponding graft end. In this work, We implemented all these steps using Python scripting.

4 Selection of Tibial Compartments

Following, we provide the mathematical formulae for selecting the TLC and TMC points, respectively. We start from selecting the most medial and lateral points of the tibia plateau. Their position is measured by the vectors $\overrightarrow{MP}$ and $\overrightarrow{LP}$ expressed in the global reference frame. The medial and lateral centers are estimated at 25 % and 75 % points of the vector $\overrightarrow{ML}$ length, as illustrated in Fig S6.

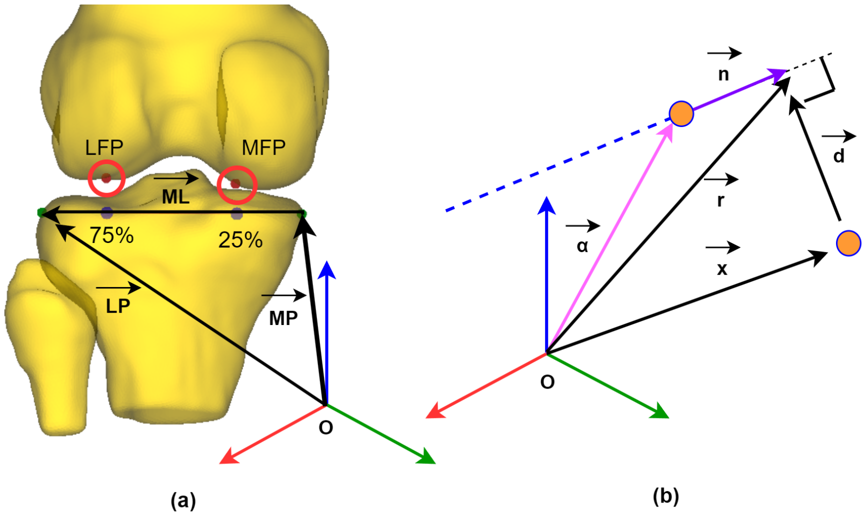

S6 Figure. Determining TLC and TMC projections on femur. a) The position of TMC and TLC are estimated at 25% and 75% of the tibia plateu width. b) A schematic presentation for estimating a point's distance from a line that is defined in vectorial form.

Then, the projections of these points on the femur mesh are estimated as the femur mesh vertices that are located in the smallest distance from a line that passes through $\overrightarrow{MP}$ and $\overrightarrow{LP}$ and has the direction of the z-axis. To estimate the distance we implement the vectorial form of line equation. For a line passing through a point $\overrightarrow{a}$ and along a direction $\hat{b}$ the line equation is: $\overrightarrow{r} = \overrightarrow{a} + t\hat{n}$.

The distance d is given by the formula:

$$||\overrightarrow{d}|| = ||\overrightarrow{x} - \overrightarrow{a} - t\hat{n}|| \quad (14)$$

The parameter t is estimated as follows:

$$
\begin{aligned}
\overrightarrow{d} \cdot \hat{n} &= 0 \\
(\overrightarrow{x} - \overrightarrow{r}) \cdot \hat{n} &= 0 \\
\overrightarrow{x} \cdot \hat{n} - \overrightarrow{r} \cdot \hat{n} &= 0 \\
\overrightarrow{x} \cdot \hat{n} - \overrightarrow{d} \cdot \hat{n} - t\hat{n} \cdot \hat{n} &= 0 \\
t = \frac{(\overrightarrow{x} - \overrightarrow{d}) \cdot \hat{n}}{||\hat{n}||^2}
\end{aligned}
$$

We applied the above formulae to the medial and lateral points and found the corresponding closest points on the femur mesh. The lateral point displacement during each simulation served as the measurement of the ATT.

5 Simulation Post - Processing

Finally, We have created scripts that parse the ".log" file generated after each successful simulation. We extract information about the position and quaternion of each rigid body reference frame for each time step relative to the initial configuration. Thus, we can create the following transformations:

- T_W_F: Transformation expressing the femur reference frame in world
- T_W_T: Transformation expressing the tibia reference frame in world

First, we multiply these transformation matrices with the initial transformation matrix that is created based on each bone reference frame acquired from OpenKnee (s) project. Thus, we have at each time step the configuration of each rigid body. Then, we create the matrix $T\_F\_T = (T\_W\_F)^{-1} * T\_W\_T$ that gives us the tibia configuration relative to the femur. From this matrix we extract ETR based on the Grood and Suntay work [5].

As we described we use the projection of the TLC center point on the femur to estimate ATT of TLC. To obtain ATT at each time step we multiply the position vector of this projection by the matrix T_W_F.

Bibliography

[1] Snehal Chokhandre, Erica E. Neumann, Tara F. Nagle, Robb W. Colbrunn, Chris A. Flask, Ceylan Colak, Jason Halloran, and Ahmet Erdemir. Specimen specific imaging and joint mechanical testing data for next generation virtual knees. *Data in Brief*, 35:106824, Apr 2021.

[2] Robb W. Colbrunn, Jarrod E. Dumpe, Tara F. Nagle, Joel D. Kolmodin, Wael K. Barsoum, and Paul M. Saluan. Robotically simulated pivot shift that represents the clinical exam. *Journal of Orthopaedic Research*, 37(12):2601–2608, Dec 2019.

[3] Lars Engebretsen, William D. Lew, Jack L. Lewis, and Robert E. Hunter. The effect of an iliotibial tenodesis on intraarticular graft forces and knee joint motion. *The American Journal of Sports Medicine*, 18(2):169–176, Mar 1990.

[4] Alan Getgood, Christopher Hewison, Dianne Bryant, Robert Litchfield, Mark Heard, Greg Buchko, Laurie A. Hiemstra, Kevin R. Willits, Andrew Firth, and Peter MacDonald. No difference in functional outcomes when lateral extra-articular tenodesis is added to anterior cruciate ligament reconstruction in young active patients: The stability study. *Arthroscopy: The Journal of Arthroscopic & Related Surgery*, 36(6):1690–1701, Jun 2020.

[5] Edward Grood and W.J. Suntay. A joint coordinate system for the clinical description of three-dimensional motions: Application to the knee. *Journal of biomechanical engineering*, 105:136–44, 06 1983.

[6] Niels Hammer, Uwe Lingslebe, Gabriela Aust, Thomas L. Milani, Carsten Hädrich, and Hanno Steinke. Ultimate stress and age-dependent deformation characteristics of the iliotibial tract. *Journal of the Mechanical Behavior of Biomedical Materials*, 16:81–86, Dec 2012.

[7] Darren Hart, Tanner Gurney-Dunlop, Jeff Leiter, Robert Longstaffe, Ahmed Shawky Eid, Sheila McRae, and Peter MacDonald. Biomechanics of hamstring tendon, quadriceps tendon, and bone–patellar tendon–bone grafts for anterior cruciate ligament reconstruction: a cadaveric study. *European Journal of Orthopaedic Surgery 'I&' Traumatology*, Apr 2022.

[8] Alexandria Mallinos, Kerwyn Jones, and Brian Davis. Pivot shift and lachman test simulation-based exploration in juvenile populations for accurately predicting anterior tibial translation. *Journal of Biomechanics*, 136:111069, May 2022.

[9] Niv Marom, Hamidreza Jahandar, Thomas J. Fraychineaud, Zaid A. Zayyad, Hervé Ouanezar, Daniel Hurwit, Andrew Zhu, Thomas L. Wickiewicz, Andrew D. Pearle, Carl W. Imhauser, and Danyal H. Nawabi. Lateral extra-articular tenodesis alters lateral compartment contact mechanics under simulated pivoting maneuvers: An in vitro study. *The American Journal of Sports Medicine*, 49(11):2898–2907, Sep 2021.

[10] Niv Marom, Hervé Ouanezar, Hamidreza Jahandar, Zaid A. Zayyad, Thomas Fraychineaud, Daniel Hurwit, Carl W. Imhauser, Thomas L. Wickiewicz, Andrew D. Pearle, and Danyal H. Nawabi. Lateral extra-articular tenodesis reduces anterior cruciate ligament graft force and anterior tibial translation in response to applied pivoting and anterior drawer loads. *The American Journal of Sports Medicine*, 48(13):3183–3193, Nov 2020.

[11] Jan-Hendrik Naendrup, Jason P. Zlotnicki, Conor I. Murphy, Neel K. Patel, Richard E. Debski, and Volker Musahl. Influence of knee position and examiner-induced motion on the kinematics of the pivot shift. *Journal of Experimental Orthopaedics*, 6(1):11, Dec 2019.

[12] Frank R. Noyes, Andrew W. Jetter, Edward S. Grood, Samuel P. Harms, Eric J. Gardner, and Martin S. Levy. Anterior cruciate ligament function in providing rotational stability assessed by medial and lateral tibiofemoral compartment translations and subluxations. *The American Journal of Sports Medicine*, 43(3):683–692, Mar 2015.

[13] Estefania Peña, Begoña Calvo, Miguel Martínez, Daniel Palanca, and Manuel doblaré. Influence of the tunnel angle in acl reconstructions on the biomechanics of the knee joint. *Clinical biomechanics (Bristol, Avon)*, 21:508–16, 07 2006.

[14] Estefania Peña, Miguel Martínez, Begoña Calvo, Daniel Palanca, and Manuel doblaré. A finite element simulation of the effect of graft stiffness and graft tensioning in acl reconstruction. *Clinical biomechanics (Bristol, Avon)*, 20:636–44, 08 2005.

[15] K. M. Quapp and J. A. Weiss. Material characterization of human medial collateral ligament. *Journal of Biomechanical Engineering*, 120(6):757–763, Dec 1998.

[16] F. E. Rowan, S. S. Huq, and F. S. Haddad. Lateral extra-articular tenodesis with acl reconstruction demonstrates better patient-reported outcomes compared to acl

reconstruction alone at 2 years minimum follow-up. *Archives of Orthopaedic and Trauma Surgery*, 139(10):1425–1433, Oct 2019.

[17] Hanno Steinke, Uwe Lingslebe, Jörg Böhme, Volker Slowik, Vickie Shim, Carsten Hädrich, and Niels Hammer. Deformation behavior of the iliotibial tract under different states of fixation. *Medical Engineering 'I&' Physics*, 34(9):1221–1227, Nov 2012.

[18] Thomas Tampere, Wouter Devriendt, Michiel Cromheecke, Thomas Luyckx, Matthias Verstraete, and Jan Victor. Tunnel placement in acl reconstruction surgery: smaller inter-tunnel angles and higher peak forces at the femoral tunnel using anteromedial portal femoral drilling—a 3d and finite element analysis. *Knee Surgery, Sports Traumatology, Arthroscopy*, 27, 11 2018.

[19] Hans Van Der Bracht, Thomas Tampere, Pieter Beekman, Alexander Schepens, Wouter Devriendt, Peter Verdonk, and Jan Victor. Peak stresses shift from femoral tunnel aperture to tibial tunnel aperture in lateral tibial tunnel acl reconstructions: a 3d graft-bending angle measurement and finite-element analysis. *Knee Surgery, Sports Traumatology, Arthroscopy*, 26(2):508–517, Feb 2018.

[20] Chao Wan, Zhixiu Hao, Zhichang Li, and Jianhao Lin. Finite element simulations of different hamstring tendon graft lengths and related fixations in anterior cruciate ligament reconstruction. *Medical and biological engineering and computing*, 55, 05 2017.

[21] Huizhi Wang, Chaohua Fang, Mingzhu Tao, Qinyi Shi, Kaixin He, and Cheng-Kung Cheng. Hourglass-shaped grafts are superior to conventional grafts for restoring knee stability and graft force at knee flexion angle of 30° following anterior cruciate ligament reconstruction: A finite element analysis. *Frontiers in Bioengineering and Biotechnology*, 10:967411, Dec 2022.

[22] Jeffrey A. Weiss, Bradley N. Maker, and Sanjay Govindjee. Finite element implementation of incompressible, transversely isotropic hyperelasticity. *Computer Methods in Applied Mechanics and Engineering*, 135(1-2):107–128, August 1996.

[23] Wikipedia contributors. Slerp — Wikipedia, the free encyclopedia. `https://en.wikipedia.org/w/index.php?title=Slerp&oldid=1177244800`, 2023. [Online; accessed 29-September-2023].
